# Predicting Complex Traits and Exposures From Polygenic Scores and Blood and Buccal DNA Methylation Profiles

**DOI:** 10.3389/fpsyt.2021.688464

**Published:** 2021-07-29

**Authors:** Veronika V. Odintsova, Valerie Rebattu, Fiona A. Hagenbeek, René Pool, Jeffrey J. Beck, Erik A. Ehli, Catharina E. M. van Beijsterveldt, Lannie Ligthart, Gonneke Willemsen, Eco J. C. de Geus, Jouke-Jan Hottenga, Dorret I. Boomsma, Jenny van Dongen

**Affiliations:** ^1^Department of Biological Psychology, Amsterdam Public Health Research Institute, Vrije Universiteit Amsterdam, Amsterdam, Netherlands; ^2^Avera Institute for Human Genetics, Sioux Falls, SD, United States

**Keywords:** DNA methylation, methylation scores, polygenic scores, multi-omics prediction, birth weight, maternal smoking, BMI, smoking

## Abstract

We examined the performance of methylation scores (MS) and polygenic scores (PGS) for birth weight, BMI, prenatal maternal smoking exposure, and smoking status to assess the extent to which MS could predict these traits and exposures over and above the PGS in a multi-omics prediction model. MS may be seen as the epigenetic equivalent of PGS, but because of their dynamic nature and sensitivity of non-genetic exposures may add to complex trait prediction independently of PGS. MS and PGS were calculated based on genotype data and DNA-methylation data in blood samples from adults (Illumina 450 K; *N* = 2,431; mean age 35.6) and in buccal samples from children (Illumina EPIC; *N* = 1,128; mean age 9.6) from the Netherlands Twin Register. Weights to construct the scores were obtained from results of large epigenome-wide association studies (EWASs) based on whole blood or cord blood methylation data and genome-wide association studies (GWASs). In adults, MSs in blood predicted independently from PGSs, and outperformed PGSs for BMI, prenatal maternal smoking, and smoking status, but not for birth weight. The largest amount of variance explained by the multi-omics prediction model was for current vs. never smoking (54.6%) of which 54.4% was captured by the MS. The two predictors captured 16% of former vs. never smoking initiation variance (MS:15.5%, PGS: 0.5%), 17.7% of prenatal maternal smoking variance (MS:16.9%, PGS: 0.8%), 11.9% of BMI variance (MS: 6.4%, PGS 5.5%), and 1.9% of birth weight variance (MS: 0.4%, PGS: 1.5%). In children, MSs in buccal samples did not show independent predictive value. The largest amount of variance explained by the two predictors was for prenatal maternal smoking (2.6%), where the MSs contributed 1.5%. These results demonstrate that blood DNA MS in adults explain substantial variance in current smoking, large variance in former smoking, prenatal smoking, and BMI, but not in birth weight. Buccal cell DNA methylation scores have lower predictive value, which could be due to different tissues in the EWAS discovery studies and target sample, as well as to different ages. This study illustrates the value of combining polygenic scores with information from methylation data for complex traits and exposure prediction.

## Introduction

Nearly all complex traits in humans are a function of their genotype and of environmental exposures, as shown by family and twin studies (1–3). DNA-based predictors of complex traits can increasingly serve to improve prediction of health outcomes and disease and to optimize risk stratification (4) and are also considered for application in social sciences and education (5, 6). Whereas, DNA-based predictors are static and solely capture genomic information, other predictors such as those based on epigenome data are dynamic and may capture both genetic and environmental information.

Polygenic scores (PGS; sometimes referred to as Polygenic Risk Scores) are defined as the weighted sum of an individual's risk alleles, or increasing alleles for a continuous trait, of a pre-selected number of single nucleotide polymorphisms (SNPs). In some areas of medicine, polygenic risk scores are already beginning to be employed to predict individual risk of disease (7–9). The PGS of an individual for a trait is calculated by multiplying, for each SNP, the number of risk alleles by a weight and then summing over all SNPs. Weights are typically estimated in a regression analysis, from a genome-wide association study (GWAS) for the trait from an independent discovery sample (typically, a large GWAS meta-analysis), and are included in the GWAS summary statistics (i.e., the estimated effect sizes, the standard errors of the estimates and the corresponding *p*-values).

This polygenic type of approach can be generalized to other omics data, including epigenomics where it results in DNA methylation scores (MS) (10), which can be described as weighted sums of the individual's methylation levels of a selected number of CpG sites. The individual's methylation levels at each CpG in an independent study population are multiplied by their corresponding weights and summed over multiple sites. Here the weights are based on summary statistics from a single or a meta-analysis epigenome-wide association study (EWAS) of the trait. By combining the effects of multiple CpG sites into a MS, a larger proportion of variance in traits is likely be explained compared to the variance that is captured by individual CpG sites. In addition to their value for prediction of complex traits and disease risk, MSs could potentially be informative as biomarkers for environmental exposures (11) or to monitor disease progression, and might be considered in association analyses in which individual CpG sites do not achieve significance or as a dimension reduction approach in interaction and mediation analyses (12, 13).

The number of genetic variants and CpG sites associated with complex traits is growing based on findings from GWAS and EWAS meta-analyses. Birth weight was associated with 60 independent signals in a multi-ancestry GWA meta-analysis, capturing up to 4.9% of the variance in birth weight in different cohorts (14), and with 914 epigenome-wide Bonferroni-significant CpGs in an EWAS meta-analysis of multiple birth cohorts with cord blood DNA methylation data (15). Body mass index (BMI) was associated with 751 SNPs in adults in the currently largest European ancestry GWAS meta-analysis, capturing ~6% of the BMI variance (16). The currently largest EWAS meta-analysis of BMI based on whole blood from adults identified association with 278 Bonferroni-significant CpGs (12). Smoking initiation was associated with 566 genetic variants in a GWAS of more than one million individuals, capturing 3.6 and 4.2% of the variance in the trait in prediction cohorts (17). A large EWAS meta-analysis of smoking identified 18,760 CpGs significantly differentially methylated in relation to current smoking in adults at a false discovery rate (FDR) of 5% from the Cohorts for Heart and Aging Research in Genomic Epidemiology (CHARGE) consortium, and 2,623 FDR significant CpGs in association with former smoking (18). EWAS meta-analyses conducted in newborns using cord blood DNA methylation data identified 6,073 CpGs with FDR significance in association with prenatal maternal smoking (19).

Attempting to capture the DNA methylation differences, previous studies have developed polygenic methylation predictors. We extensively reviewed the literature on studies that report methylation predictors as single MS and studies that examined the combined predictive value of MS and PGS (see Supplementary Table 1). Taking the results from EWASs into independent target samples in which MSs are defined, has yielded promising results for birth weight (20), BMI (20–22), prenatal maternal smoking (23, 24), and smoking status (11, 23, 25–28). Reed et al. (20) computed MSs for birth weight based on the 135 CpGs from an adult BMI EWAS in the Framingham Heart Study and Lothian Birth cohorts (*N* = 3,743) (29). These scores captured 2% of birth weight variation in 823 ALSPAC newborns with DNA methylation data in cord blood, which was higher than the variance captured by a PGS (0.4%). Several studies created whole blood DNA MSs of BMI and made predictions in children and adults. MSs based on 78 probes from 2,377 adults of the Framingham Heart Study and weights (effect sizes) from 750 adults of the LifeLines DEEP study explained 11% of the variance in BMI in 1,366 adults from Lothian Birth cohorts and 5% of BMI variance in 403 adolescents from Brisbane Systems Genetic Study (BSGS) (21). MSs based on 400 CpGs from 2,562 Generation Scotland participants explained 10% of BMI variance in 892 adults from Lothian Birth cohort (22). MSs based on 135 probes from 3,742 adults from both Framingham Heart Study and Lothian Birth cohorts explained 10% of BMI variance in 726 ALSPAC women and up to 3% of BMI variance in children at different ages (20). It has been shown that MS for BMI perform better in adults compared to children and adolescents (20, 21). Attempts of cross-tissue performance testing were scarce (25, 30), however, it have been shown that some alterations persist across tissue types (31).

For prenatal maternal smoking, MS based on weights from cord blood DNA methylation EWASs of 1,057 newborns from Norwegian Mother and Child Cohort Study (MoBa) was tested on another MoBa subset of 221 newborns (24), and MS based on weights from cord blood DNA methylation EWAS meta-analysis of 6,685 newborns done by Joubert and colleagues (19) was tested on 754 ALSPAC women around 30 years old (23); the predictive accuracy (the amount of variation in the outcome explained by the score) was lower in women than in newborns. Smoking predictors have been described based on different numbers of probes from whole blood DNA methylation studies. Only 2 CpGs were included the smoking MS of Zhang et al. that predicted smoking status in 9,949 older adults (28). The largest smoking MSs included 2,623 Bonferroni significant CpGs from EWAS meta-analysis of 15,907 individuals (18) and predicted smoking status during pregnancy in 754 women by Richmond et al. (23). The same CpGs were used by Sugden et al. (11) to predict smoking status in 1,037 adults from the Dunedin Longitudinal Study and 2,232 twins from the Environmental Risk Longitudinal Study.

Despite the growing number of cohorts that have both genomic and methylation data, few attempts have been made to combine PGS and MS in a multi-omics model. To the best of our knowledge, BMI, and height are currently the only traits for which the prediction by PGS and MS combined has been investigated (21, 22). In a combined model, the PGS and MS together explained 17% of the variance in BMI in 1,366 adults (21) and 18% in 889 adults (22), both from the Lothian Birth cohorts, 13–16% in 750 adults from Lifelines and 8% in adolescents from the Brisbane Systems Genetic Study (21), corresponding to an added ~4–9% extra variance explained compared to the PGS alone.

We expand on the previous work by addressing several points. First, it is largely unknown to what extent MS based on EWAS weights derived in adults predict trait variation in children and vice versa. Second, previous studies of MS were based on cord blood or whole blood, and it is unknown if these scores translate to other tissues. Third, for all traits, except BMI and height (20, 21), it is unknown whether MS add to prediction independently of PGS.

In the current biomarker study, we analyze the predictive accuracy of PGS and MS (both individually and combined). The goal of our study is to examine if the MSs add predictive value above the PGSs. The weights required for DNA methylation data were obtained large EWAS and applied to methylation levels from two different tissues (blood and buccal). We analyze data from large groups of adults with DNA methylation in blood (*N* = 2,431, mean age = 35.6) and children with DNA methylation in buccal cells (*N* = 1,128, mean age = 9.6) who participate in research projects of the Netherlands Twin Register and consider multiple traits. For an early-life trait we analyze birth weight, and for a trait that is dynamic in childhood and adulthood, we analyze BMI. As early and later life exposures we examine prenatal maternal smoking during pregnancy and own smoking. These four phenotypes represent complex traits and exposures with different relative contributions of genetics and environment to inter-individual variance.

## Materials and Methods

### Overview

This study included adults and children who participated in studies from the Netherlands Twin Register (NTR). DNA samples in adult twins and family members were isolated from whole blood DNA data and in twin children from buccal cells. Adults took part in the NTR-Biobank (32) and children in the FP7-Action project (33–36). The study was approved by the Central Ethics Committee on Research Involving Human Subjects of the VU University Medical Centre, Amsterdam, an Institutional Review Board certified by the U.S. Office of Human Research Protections (IRB number IRB00002991 under Federal-wide Assurance FWA00017598; IRB/institute codes, NTR 03-180). Adults provided written informed consent, for children consent was given by their parents.

### Adults

#### Study Population and Samples

After quality control, genome-wide DNA methylation profiles in whole blood and genotype data were available for 2,431 NTR adults (37). This dataset included 2,426 individuals from twin pairs, and 5 family members (mothers and spouses). The mean age at DNA collection was 35.6 years (range = 17.6–79.2 years) and 32.7% of subjects were males. For 20 participants, longitudinal methylation data (methylation data at two time points) were available. Individuals with missing data on phenotypes or covariates, and phenotype outliers were excluded from analysis, resulting in a sample size of 2,040 for birth weight, 2,410 for BMI, 1,914 for current vs. never smoking, and 1,938 for former vs. never smoking. Because prenatal maternal smoking exposure is equal for co-twins, one twin from each pair was randomly included in the analysis, resulting in a sample size of 720. The blood sampling procedure has been described by Willemsen et al. (32).

#### DNA Methylation

DNA methylation in blood was assessed with the Infinium HumanMethylation450 BeadChip Kit (Illumina, San Diego, CA, USA) by the Human Genotyping facility (HugeF) of ErasmusMC, the Netherlands (http://www.glimdna.org/) as part of the Biobank-based Integrative Omics Study (BIOS) consortium (38). DNA methylation measurements have been described previously (37, 38). Genomic DNA (500 ng) from whole blood was bisulfite treated using the Zymo EZ DNA Methylation kit (Zymo Research Corp, Irvine, CA, USA), 12 μl of buffer was utilized to elute the converted DNA off the column after conversion, and 4 μl (~33 ng/μl) of bisulfite-converted DNA was measured on the Illumina 450 K array following the manufacturer's protocol. A number of sample- and probe-level quality checks and sample identity checks were performed, as described in detail previously (37). In short, sample-level QC was performed using MethylAid (39). Probes were set to missing in a sample if they had an intensity value of exactly zero, or a detection *p* > 0.01, or a bead count of <3. After these steps, probes that failed based on the above criteria in >5% of the samples were excluded from all samples (only probes with a success rate ≥0.95 were retained). For all samples, ambiguously mapped probes were excluded, based on the definition of an overlap of at least 47 bases per probe from Chen et al. (40), and all probes containing a SNP, identified in the Dutch population (41), within the CpG site (at the C or G position) were excluded, irrespective of minor allele frequency. Only autosomal sites were kept in the current analyses (*N* = 411,169). The methylation data were normalized with functional normalization (42). Probes with missing values (probes with missing values in more than 5% of the sample were removed) were imputed with the function imputePCA from the package missMDA as implemented in the pipeline for DNA methylation array analysis developed by the Biobank-based Integrative Omics Study (BIOS) consortium (43).

#### Phenotyping

Data on birth weight were obtained from self-report or by parental report. If data were available from multiple surveys by Adult Netherlands Twin Register (ANTR) and/or informants, they were checked for consistency (44). When multiple data points differed by <200 g, the average was taken, and in the cases of larger differences, data were excluded. Information on maternal smoking during pregnancy was obtained in ANTR Survey 10 (data collection in 2013) with the following question: “Did your mother ever smoke during pregnancy?” Answer categories were “no,” “yes,” and “I don't know.” For twin pairs, the answers were checked for consistency and missing data for one twin were supplemented with data from the co-twin where possible. In the case of inconsistent answers, the data from both co-twins were set to missing. If both twins answered “I don't know,” the variable was coded as missing. Data on body mass index (BMI) and smoking status were collected at blood draw (32). We analyzed two smoking phenotypes: current smokers (1) vs. never smokers (0), and former smokers (1) vs. never smokers (0). The percentage of white blood cell was obtained in fresh blood samples collected in EDTA (Ethylene Diamine Tetra Acetic acid) tubes (45). For birth weight and BMI, we removed outliers using a cut-off of 3 standard deviations from the mean. For birth weight, 6 outliers were removed; for BMI, 27 outliers were removed.

### Children

#### Study Population and Samples

Genotype data and genome-wide DNA methylation profiles in buccal cells were collected in a children that participated in a larger project on childhood aggression “Aggression in Children: Unraveling gene-environment interplay to inform Treatment and InterventiON strategies” (ACTION; http://www.action-euproject.eu/) and consists of twins who score high or low on aggression (33–36). After quality control, genome-wide DNA methylation data and genotype data were available for 1,128 children from twin pairs (mainly monozygotic twins). The mean age at DNA collection was 9.6 years (range = 5.6–12.9 years) and 52.8% were males. For 2 participants, a technical replicate measure on with the Infinium MethylationEPIC BeadChip Kit was included (36). Individuals without missing data on phenotypes or covariates were included in the analyses, and phenotype outliers were excluded, resulting in a sample size of 1,070 children for birth weight and 1,072 for BMI. Because prenatal maternal smoking exposure is equal for co-twins, one twin from each pair was randomly included in the analysis, resulting in a sample size of 547. The sample collection protocol is available at: http://www.action-euproject.eu/content/data-protocols. DNA was collected from buccal swabs at home: 16 cotton sticks were individually rubbed against the inside of the cheek in the morning and evening on 2 days by the participants and placed in buffer. Individuals were asked to refrain from eating or drinking 1 h prior to sampling. High molecular weight genomic DNA was extracted from the swabs by standard DNA extraction techniques and visualized using agarose gel electrophoresis. The DNA samples were quantified using the Quant-iT PicoGreen dsDNA Assay Kit (ThermoFisher Scientific, Waltham, MA, USA).

#### DNA Methylation

DNA methylation was assessed with the Infinium MethylationEPIC BeadChip Kit (Illumina, San Diego, CA, USA) by the Human Genotyping facility (HugeF) of ErasmusMC, the Netherlands (http://www.glimdna.org/) [see van Dongen et al. (36)]. Quality control (QC) and normalization of the methylation data were performed using a pipeline developed by the Biobank-based Integrative Omics Study (BIOS) consortium (43), which includes sample quality control using the R package MethylAid (39) and probe filtering and functional normalization (42) as implemented in the R package DNAmArray. The following probe filters were applied: probes were set to missing (NA) in a sample if they had an intensity value of exactly zero, detection *P* > 0.01, or bead count <3; probes were excluded from all samples if they mapped to multiple locations in the genome, if they overlapped with a SNP or Insertion/Deletion (INDEL), or if they had a success rate <0.95 across samples. Annotations of ambiguous mapping probes (based on an overlap of at least 47 bases per probe) and probes where genetic variants (SNPs or INDELS) with a minor allele frequency > 0.01 in Europeans overlap with the targeted CpG or single base extension site (SBE) were obtained from Pidsley et al. (46). For two twins, a technical replicate measure on EPIC was obtained (on different BeadChip Arrays). Probes with missing values (probes with missing values in more than 5% of the sample were removed) were imputed with the function imputePCA from the package missMDA as implemented in the pipeline for DNA methylation array analysis developed by the BIOS consortium (43).

#### Phenotyping

Data on birth weight of the young twins came from surveys sent to mothers shortly after the registration of the newborn twins (47). Data on BMI were collected from surveys filled out by mothers and fathers in the Young Netherlands Twin Register (YNTR) when children were around 5, 7, 10, and 12 years of age. If both parents completed the survey, preference was given to data provided by the mother. BMI closest to the date of DNA collection was selected. The average time between DNA collection and BMI assessment was 1.9 years before the survey (median = −0.9, range: from buccal sample collection 10.3 years before survey to buccal sample collection 2.1 years after survey). Information on maternal smoking during pregnancy was reported by mothers after registration for three trimesters of pregnancy and was coded as “non-smoking” if the mother did not smoke during the entire pregnancy and “smoking” if the mother smoked at least during one trimester (48). For birth weight and BMI, we removed outliers using a cut-off of 3 standard deviations from the mean. For birth weight, 1 outlier was removed; for BMI, 12 outliers were removed.

Cellular proportions were predicted with hierarchical epigenetic dissection of intra-sample-heterogeneity (HepiDISH) with the RPC method (reduced partial correlation), as described by Zheng et al. (49) and implemented in the R package HepiDISH. HepiDISH is a cell-type deconvolution algorithm developed for estimating cellular proportions in epithelial tissues based on genome-wide methylation profiles and makes use of reference DNA methylation data from epithelial cells, fibroblasts, and seven leukocyte subtypes. This method was applied to the data after data QC and normalization.

### Genotyping

Genotyping in children (YNTR) and adults (ANTR) was done on multiple platforms over time including Perlegen-Affymetrix, Affymetrix 6.0, Affymetrix Axiom, Illumina Human Quad Bead 660, Illumina Omni 1M and Illumina GSA. Quality control and processing of the genotype data was performed on the complete dataset of all genotyped participants from the NTR. Quality control was carried out and haplotypes were estimated in PLINK. CEU population outliers, based on per platform 1000 Genomes PC projection with the Smartpca software (50), were excluded. Data were phased per platform using Eagle, and then imputed to 1000 Genomes using Minimac, following the Michigan imputation server protocols. For the polygenic scoring imputed data were converted to best guess genotypes, and filtered to include only ACGT SNPs, SNPs with MAF > 0.01, HWE *p* > 10^−5^ and genotype call rate > 0.98, and exclude SNPs with more than 2 alleles. All Mendelian errors were set to missing. Principal components (PCs) were calculated with Smartpca using linkage-disequilibrium-pruned (LD-pruned) 1000 Genomes–imputed SNPs that were also genotyped on at least one platform, had MAF > 0.05 and were not present in the long-range LD regions.

### Statistical Methods

#### EWAS and GWAS Summary Statistics

MSs and PGSs were created using weights based on large epigenome-wide association study (EWAS) and genome-wide association study (GWAS) meta-analyses. These studies are summarized in Table 1. Additional information on the studies and derived scores is provided in Supplementary Tables 2, 3.

**Table 1:** Discovery epigenome-wide and genome-wide association studies that provided the summary statistics to calculate DNA methylation scores and polygenic scores.

**Trait/Exposure**	**References**	**Phenotype in Discovery Study—DNA methylation Tissue**	***N* discovery cohort used to create scores**	***N* CpGs/SNPs reported at significant level in reference**
**EWAS**				
Birth weight	Küpers et al. (15)	Birth weight—Cord blood	8,825 newborns	914 (Bonferroni)
BMI	Wahl et al. (12)	BMI—Whole blood	5,387 adults	278 (Bonferroni)
Prenatal maternal smoking	Sidkar et al. (51)^*^	Prenatal maternal smoking—Cord blood	4,994 newborns (897 exposed to sustained prenatal smoking)	5,547 (FDR <0.05)
Smoking	Sidkar et al. (51)^*^	Current vs. never smoked—Whole blood	9,389 adults (2,433 current smokers)	34,541 (FDR <0.05)
	Joehanes et al. (18)	Former vs. never smoked—Whole blood	13,474 adults (6,518 former smokers)	2,623 (FDR <0.05)
**GWAS**				
Birth weight	http://www.nealelab.is/uk-biobank/	Birth weight	280,250 (UK biobank)	not published
BMI	Yengo et al. (16)	BMI	~700,000 individuals of different ancestry 652,099^**^ were used in our study for calculation of PGS	941
Prenatal maternal smoking	UK Biobank http://www.nealelab.is/uk-biobank/	Maternal smoking around birth	331,862 (UK Biobank)	Not published
Smoking	Liu et al. (17)	Smoking initiation (ever/never smoked)	Up to 1.2 million individuals in discovery study 625,536^**^ used in our study for PGS	566

*EWAS, Epigenome-wide association study; GWAS, Genome-wide association study; FDR, False Discovery Rate*.

* *Sikdar et al. (51) repeated the meta-analysis by Joubert et al. (2016) (EWAS in newborns) and Joehanes et al. (18) (EWAS in never vs. current smokers), and provided full genome-wide summary statistics for a fixed-effects meta-analysis of maternal smoking in newborns (cord blood) and for current vs. never smokers (whole blood)*.

** *NTR and 23andMe are excluded. For additional details, For additional details, see Supplementary Tables 2, 3*.

#### DNA Methylation Scores

The effect sizes obtained from the summary statistics from previously published EWAS meta-analyses (Table 1, Supplementary Table 2) were used to calculate weighted MSs in NTR participants as previously done by Sugden et al. (11), Wahl et al. (12), Shah et al. (21), Richmond et al. (23), and Elliott et al. (26). For each trait and for each individual, a score was calculated by multiplying the methylation level at a given CpG by the previously reported effect size of the CpG (β), and then summing these values over all CpGs:

$$\begin{array}{l} DNA\;methylation\;score=\beta_{1}^{*}CpG_{1}+\beta_{2}^{*}CpG_{2}\ldots .+\;\beta_{\text{i}}^{*}CpG_{\text{i}} \end{array}$$

where CpG_i_ is the methylation level at CpG site *i*, which ranges between 0 and 1, and β_i_ is the effect size (regression coefficient) at the CpG_i_ obtained from summary statistics of EWAS meta-analyses that did not include participants from the NTR.

For each phenotype, except for former smoking, we calculated multiple MSs based on different subsets of CpGs according to their significance level. Subsets of CpGs were selected based on *p*-value thresholds of <1 × 10^−1^, <1 × 10^−5^, and <1 × 10^−7^. For former vs. never smoking, genome-wide summary statistics were not available, and we calculated MSs for former smoking based on CpGs that were significant in the EWAS of former vs. never smokers at a False Discovery Rate of 5%. Additionally, we tested prediction of former smoking based on the MSs derived from the genome-wide EWAS summary statistics of current vs. never smoking. To examine if removal of CpGs with correlated DNA methylation levels affects trait prediction, we also calculated pruned scores by step-wise selection of the most significant CpG site and excluding CpG sites with a correlation of 0.1 or higher [threshold chosen based on Shah et al. (21)] in order to keep an independent set of CpGs.

#### Polygenic Scores

Polygenic scores (PGSs) were calculated based on weighting of genotypes by effect sizes as made available from GWAS summary statistics (see Table 1, Supplementary Table 3) in discovery samples without NTR. Before calculating the PGSs, linkage disequilibrium (LD) weighted β's were calculated from these summary statistics by the LDpred package to correct for the effects of LD and to maximize predictive accuracy of the PGSs (52). QC has been applied: MAF > 0.01, duplicated SNPs, mismatching alleles, ambiguous SNPs were excluded. We randomly selected 2,500 unrelated individuals from NTR as a reference population to calculate the LD patterns. The adjusted β's were calculated from an LD pruning window of 250 KB, with the fraction of causal SNPs set at 0.01 for birth weight, because this fraction was previously shown to perform optimally for birth weight in the NTR population (53) and at 0.50 for other phenotypes. The PGSs were obtained for all NTR participants with genotyping data with the PLINK 1.9 software.

#### Statistical Analysis

Continuous traits, MSs and PGSs were z-score transformed [trait value—trait mean/trait standard deviation] before analysis. Pairwise Pearson correlations between each trait, MSs, PGSs, and covariates were computed in NTR adults and children EWAS datasets for each phenotype and visualized in correlation plots. For each trait, we fitted a series of regression models to examine: (1) the predictive value of MSs; (2) the predictive value of a PGS; and (3) whether MS and PGS contributed independently to trait prediction in a combined predictor. First, for each trait, we evaluated the performance of multiple different MSs based on different *p*-value thresholds, pruned, and unpruned. We took the score that explained the largest amount of variance forward to the combined model. Second, we evaluated the performance of PGS in prediction of each trait. Finally, we examined if MSs predict these traits over and above the PGSs and estimated how much variance in each trait was explained by multi-omics predictor, e.g., by MSs and PGSs together. Sex and age at DNA collection were included as covariates in all three models. In the prediction models with whole blood DNA MSs, we corrected for percentages of neutrophils, monocytes, and eosinophils. In the prediction models with buccal DNA MSs, we corrected for epithelial cell and natural killer cell proportions. To adjust for technical variation, array row and bisulfite plate (dummy-coding) were included as covariates in all models with EWAS covariates. In models including PGSs, we corrected for genotype data-specific covariates: the first ten genetic principal components and genotype platform dummy variables (GWAS covariates).

##### Continuous Traits

For birth weight and BMI, the following models were fitted in each of the two datasets (whole blood methylation data from adults and buccal methylation data from children):

Model 1: Trait ~ MS + sex + age + EWAS covariates
Model 2: Trait ~ PGS + sex + age + GWAS covariates
Model 3: Trait ~ MS + PGS + sex + age + EWAS covariates + GWAS covariates

Analyses were carried out with generalized estimation equation (GEE) models accounting for familial relatedness, fitted with the R package “gee” with the following settings: Gaussian link function for continuous data, 100 iterations, and the “exchangeable” option to account for the correlation structure within families. To calculate the variance explained by the MS and the PGS, we squared the regression coefficient of each score obtained in GEE. This value was multiplied by 100 to obtain the percentage of variance explained.

##### Dichotomous Traits

For dichotomous traits, i.e., prenatal maternal smoking, current vs. never smoking, and former vs. never smoking, the following models were fitted in two datasets (whole blood methylation data from adults and buccal methylation data from children):

Model 1a: Trait ~ MS + sex + age + EWAS covariates
Model 1b: Trait ~ sex + age + EWAS covariates
Model 2a: Trait ~ PGS + sex + age + GWAS covariates
Model 2b: Trait ~ sex + age + GWAS covariates
Model 3a: Trait ~ MS + PGS + sex + age + EWAS covariates + GWAS covariates
Model 3b: Trait ~ PGS + sex + age + EWAS covariates + GWAS covariates
Model 3c: Trait ~ MS + sex + age + EWAS covariates + GWAS covariates

To obtain the variance explained, models were fitted with logistic regression with binomial family setting (link = “logit”). Estimation of the variance explained by the MS and PGS, was based on the approach proposed by Lee et al., where coefficients of determination (*R*^2^) for binary responses are calculated on the liability scale (54). *R*^2^ is equal to the explained variance divided by the total variance; that is the sum of explained variance and residual (homoscedastic) variance. We first regressed the trait on the MS, sex, age and EWAS covariates (model 1a), and then on sex, age, and EWAS covariates only (model 1b). We calculated variance explained by all predictors in each model. We calculated the predictive value of the MS by subtracting the difference between the variance explained by the model 1a and 1b. The same was done for models with PGS with sex, age, and GWAS covariates (model 2a and 2b), and then for combined model with both MS and PGS scores (models 3a-c). In the last case, the difference between explained variance in model 3a and model 3b gave us an estimate explained by MS, and the difference between explained variance in model 3a and model 3c resulted in estimate explained by PGS.

To correct for relatedness in smoking prediction, *p*-values were obtained from GEE models, fitted with the R package “gee,” with the binomial link function for dichotomous data, 100 iterations, and the “exchangeable” option to account for the correlation structure within families. For prenatal smoking exposure (yes/no) we randomly chose one of the twins from the pair, and *p*-values were obtained from logistic regression models.

#### Sensitivity Analysis

We carried out a sensitivity analysis in which we repeated the models for BMI prediction in children from MSs after removal of children for whom information on BMI was collected more than 3 years before or after DNA collection (*N* = 324 children removed; new *N* = 748).

#### Multiple Testing Correction

Statistical significance was assessed following Bonferroni correction for multiple testing (six tests in model 1 for birth weight, BMI, prenatal maternal smoking and current smoking, seven tests in model 1 for former smoking, one test in models 2 and 3 for each trait in adults; the same number of tests in children except smoking status and plus eight tests in sensitivity analysis for BMI). This resulted in a significance level of 0.0012 (α = 0.05/42) for adults and 0.0016 (α = 0.05/32) for children.

## Results

**Figure 1:**
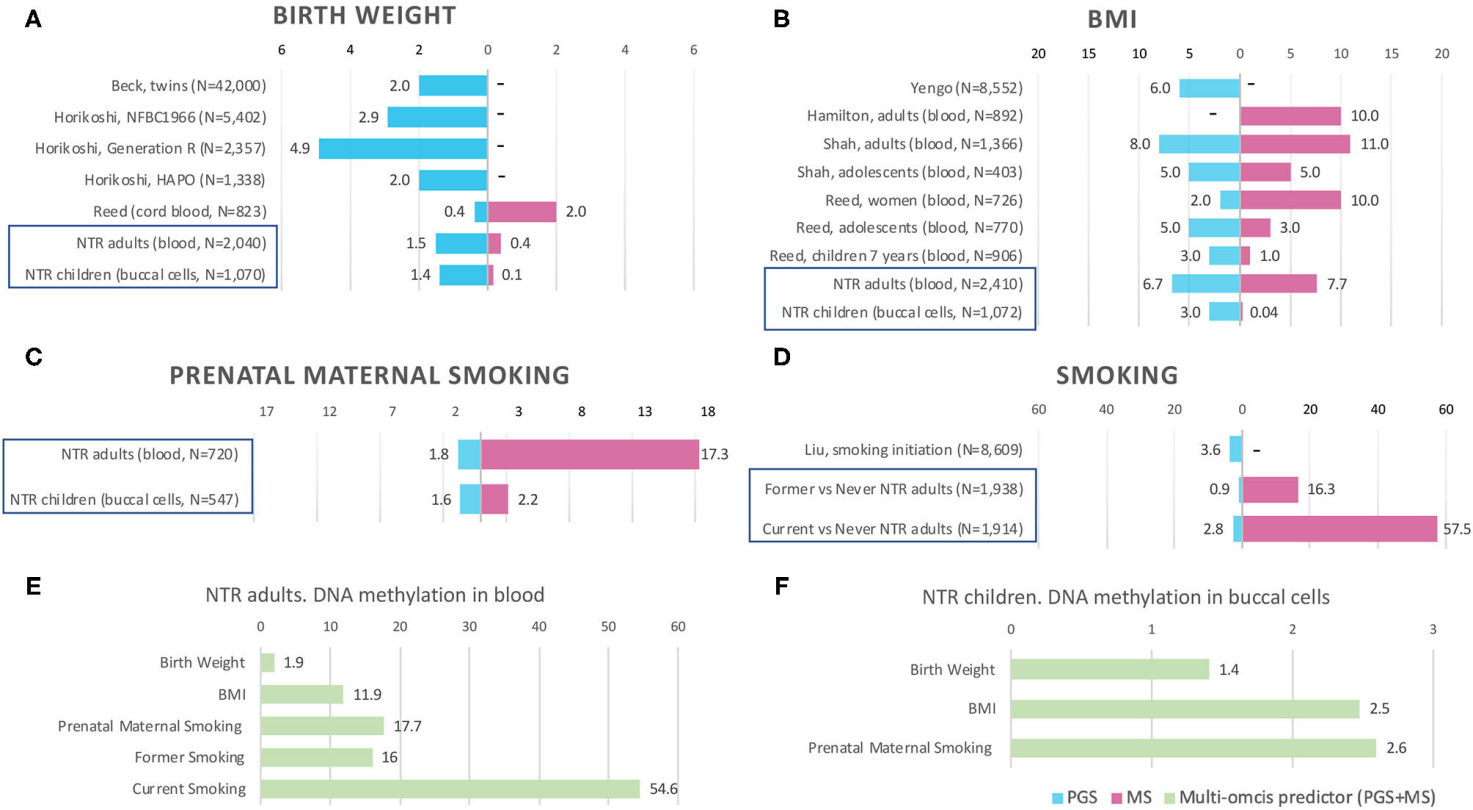
Prediction by methylation and polygenic scores in previous studies and NTR. Bars are the phenotypic variance explained by the score (*R*^2^), x-axis shows *R*^2^ in %. MS, methylation score. PGS, polygenic score. PGS+MS, polygenic and methylation scores in combined model (multi-omics predictor). “–” indicates that the score is not available in the study. Prediction by PGS and MS separately in NTR cohorts is indicated by blue frames in **(A–D)**. Multi-omics prediction in NTR is presented in **(E–F)**. Full references on previous studies in **(A,B,D)** can be found by first author in References. For more details on previous EWASs included in **(A,B)**, see Supplementary Table 1.

Characteristics of the NTR adult and children are presented in Table 2. Distribution of the traits/exposures as main outcomes and MSs and PGs as predictors are presented in Supplementary Figures 1–3. Correlations between trait/expoosure, PGS, MSs, sex, age, and cellular compositions of the samples are shown in Supplementary Figures 4, 5. The correlations between PGS and MSs for the same trait were weak in adults (*r* = [0.01–0.15]) and children (*r* = [0.01–0.05]). Further, we report the correlation between the PGS and the MS that captured the largest amount of variation in the trait. We examined prediction of each phenotype by its MS and PGS separately. The explained variance and corresponding *p*-values for unpruned and pruned MSs with different thresholds for inclusion of CpGs are presented in Table 3, and for PGS in Table 4. To examine to what extent the PGS and the MS capture independent information, we fitted the model in which the outcome was regressed on both scores as multi-omics prediction presented in Table 5. Figure 1 shows the variance explained by the MSs and PGSs separately and together as multi-omics predictor in previous and our studies.

**Table 2:** Characteristics: NTR adults and children.

	**Adults**	**Children**
**DNA methylation tissue**	**Whole blood**	**Buccal cells**
DNA methylation array	Illumina 450 k	Illumina EPIC
Sample size	2,431	1,128
Age, mean (sd)	35.6 (11.9)	9.6 (1.9)
Males, *n* (%)	794 (32.7%)	596 (52.8%)
Females, *n* (%)	1,637 (67.3%)	532 (47.2%)
Birth weight, mean (sd)	2,507.8 (573.7)	2,395.9 (544.1)
Missing	404	59
BMI, mean (sd)	24.1 (3.9)	16 (2.1)
Missing	14	46
Prenatal maternal smoking (no), *n* (%)	1,169 (90.1%)	921 (91.1%)
Prenatal maternal smoking (yes), *n* (%)	129 (9.9%)	90 (8.9%)
Missing	1,133	117
Never smokers, *n* (%)	1,395 (57.5%)	NA
Former smokers, *n* (%)	527 (21.7%)	NA
Current smokers, *n* (%)	506 (20.8%)	NA
Missing	3	NA
**Cell counts, mean (sd)**		
Neutrophil percentage	52.5 (9.1)	NA
Eosinophil percentage	3.1 (2.3)	NA
Monocyte percentage	8.4 (2.4)	NA
Epithelial cell proportion	NA	0.806 (0.116)
NK cell proportion	NA	0.03 (0.013)

*NTR, Netherlands Twin Register; sd, standard deviation; NA, not available; NK, natural killer cells*.

*Descriptives are provided for covariates that were included as covariates in the prediction models*.

*The number of samples with complete data on DNA methylation, cell counts, and genotyping data are presented. For a small number of individuals, two samples are included in the analysis in adults (n = 20) and children (n = 2)*.

**Table 3:** Results of the methylation score prediction of birth weight, BMI, prenatal maternal smoking and current and former smoking.

**Trait**	**Group—DNA methylation tissue**	**CpGs included in MS**	**N CpGs**	**β_MS_**	**SE_**MS**_**	**P_**MS**_**	**MS *R*^**2**^ (%)**
Birth weight	Adults—Whole blood (*N* = 2,040)	*p* < 10^−1^	72,570	0.044	0.029	0.127	0.192
		*p* < 10^−5^	2,274	0.055	0.029	0.057	0.304
		*p* < 10^−7^	963	0.049	0.027	0.074	0.238
		*p* < 10^−1^ pruned^**^	934	0.062	0.022	0.004	0.386
		*p* < 10^−5^ pruned	30	0.015	0.023	0.513	0.023
		*p* < 10^−7^ pruned	18	0.025	0.023	0.277	0.064
	Children—Buccal cells (*N* = 1,070)	*p* < 10^−1^	72,205	−0.013	0.031	0.679	0.016
		*p* < 10^−5^	2,249	0.031	0.032	0.344	0.094
		*p* < 10^−7^^**^	958	0.038	0.032	0.237	0.141
		*p* < 10^−1^ pruned	184	0.015	0.029	0.613	0.022
		*p* < 10^−5^ pruned	13	0.016	0.032	0.613	0.025
		*p* < 10^−7^ pruned	9	−0.005	0.033	0.882	0.002
BMI	Adults—Whole blood (*N =* 2,410)	*p* < 10^−1^	55,653	0.134	0.025	6.98 × 10^−08^^*^	1.786
		*p* < 10^−5^	1,067	0.261	0.026	5.07 × 10^−24^^*^	6.822
		*p* < 10^−7^^**^	412	0.277	0.026	9.79 × 10^−27^^*^	7.673
		*p* < 10^−1^ pruned	671	0.124	0.021	3.14 × 10^−09^^*^	1.538
		*p* < 10^−5^ pruned	13	0.220	0.023	1.70 × 10^−22^^*^	4.827
		*p* < 10^−7^ pruned	6	0.206	0.023	7.50 × 10^−20^^*^	4.258
	Children—Buccal cells (*N* = 1,072)	*p* < 10^−1^	55,279	0.003	0.017	0.878	0.001
		*p* < 10^−5^	1,079	−0.006	0.017	0.733	0.003
		*p* < 10^−7^	422	−0.008	0.018	0.676	0.006
		*p* < 10^−1^ pruned^**^	183	0.021	0.019	0.276	0.042
		*p* < 10^−5^ pruned	13	0.005	0.019	0.772	0.003
		*p* < 10^−7^ pruned	6	0.007	0.018	0.720	0.004
Prenatal maternal smoking	Adults—Whole blood (*N* = 720)	*p* < 10^−1^	76,531	0.761	0.168	5.83 × 10^−06^^*^	8.512
		*p* < 10^−5^	1,581	0.946	0.140	1.51 × 10^−11^^*^	15.115
		*p* < 10^−7^^**^	607	1.009	0.136	1.28 × 10^−13^^*^	17.277
		*p* < 10^−1^ pruned	962	0.580	0.136	2.01 × 10^−05^^*^	7.076
		*p* < 10^−5^ pruned	33	0.661	0.123	7.12 × 10^−08^^*^	6.820
		*p* < 10^−7^ pruned	16	0.620	0.119	1.94 × 10^−07^^*^	5.963
	Children—Buccal cells (*N* = 547)	*p* < 10^−1^	76,146	−0.256	0.158	0.105	0.930
		*p* < 10^−5^	1,571	−0.285	0.187	0.127	1.683
		*p* < 10^−7^^**^	606	−0.304	0.183	0.096	2.223
		*p* < 10^−1^ pruned	187	−0.125	0.153	0.416	0.386
		*p* < 10^−5^ pruned	16	−0.096	0.164	0.559	0.149
		*p* < 10^−7^ pruned	9	−0.120	0.161	0.456	0.274
Smoking current vs. never	Adults—Whole blood (*N =* 1,914)	*p* < 10^−1^	98,972	1.420	0.095	4.65 × 10^−43^^*^	20.974
		*p* < 10^−5^	11,433	1.923	0.104	1.93 × 10^−66^^*^	40.086
		*p* < 10^−7^	6,938	1.999	0.105	6.89 × 10^−69^^*^	44.449
		*p* < 10^−1^ pruned	913	1.170	0.070	2.70 × 10^−47^^*^	27.626
		*p* < 10^−5^ pruned	37	2.194	0.108	5.21 × 10^−75^^*^	56.237
		*p* < 10^−7^ pruned^**^	24	2.245	0.111	1.25 × 10^−73^^*^	57.461
Smoking former vs. never	Adults—Whole blood (*N* = 1,938)	*p* < 10^−1^ from current vs. never smoking EWAS	98,972	0.429	0.085	6.63 × 10^−07^^*^	2.004
		*p* < 10^−5^ from current vs. never smoking EWAS	11,433	0.689	0.086	2.73 × 10^−15^^*^	5.127
		*p* < 10^−7^ from current vs. never smoking EWAS	6,938	0.758	0.086	3.37 × 10^−18^^*^	6.217
		FDR significant from Former vs. Never Smoking EWAS	2,568	0.935	0.088	3.56 × 10^−24^^*^	9.340
		*p* < 10^−1^ pruned from current vs. never smoking EWAS	913	0.499	0.066	3.38 × 10^−13^^*^	4.399
		*p* < 10^−5^ pruned from current vs. never smoking EWAS	37	1.186	0.087	6.64 × 10^−36^^*^	15.625
		*p* < 10^−7^ pruned from current vs. never smoking EWAS^**^	24	1.226	0.088	5.44 × 10^−36^^*^	16.316
		FDR significant from Former vs. Never Smoking EWAS, pruned	2,330	0.692	0.071	1.96 × 10^−18^^*^	7.569

*β is the regression coefficient for each methylation score (MS) with standard error (SE) and p-value (P). MS R^2^ is the phenotypic variance explained by the MS*.

* *indicates p < 0.0012 in adults and <0.0016 in children*.

** *indicate methylation score with lowest p-value for a trait/exposure*.

**Table 4:** Results of the polygenic score prediction of birth weight, BMI, prenatal maternal smoking, and current and former smoking.

**Trait**	**Group**	**β_PGS_**	**SE_**PGS**_**	**P_**PGS**_**	**PGS *R*^**2**^ (%)**
Birth weight	Adults (*N* = 2,040)	0.123	0.024	1.56 × 10^−07^^*^	1.520
	Children (*N* = 1,070)	0.118	0.030	9.67 × 10^−05^^*^	1.387
BMI	Adults (*N* = 2,410)	0.259	0.022	3.43 × 10^−32^^*^	6.725
	Children (*N* = 1,072)	0.173	0.041	2.20 × 10^−05^^*^	3.003
Prenatal maternal smoking	Adults (*N* = 720)	0.259	0.132	0.049	1.797
	Children (*N* = 547)	0.282	0.165	0.086	1.622
Smoking current vs. never	Adults (*N* = 1,914)	0.330	0.056	2.24 × 10^−07^^*^	2.794
Smoking former vs. never	Adults (*N* = 1,938)	0.197	0.058	0.001	0.909

*β is the regression coefficient for each polygenic score (PGS) with standard error (SE) and p-value (P). PGS R^2^ is the phenotypic variance explained by the PGS*.

*PGS to predict current vs. never smoking and former vs. never smoking were created based on GWAS on smoking initiation (17)*.

* *indicates p < 0.0012 in adults and <0.0016 in children*.

**Table 5:** Results of the multi-omics prediction of birth weight, BMI, prenatal maternal smoking, and current and former smoking.

**Trait**	**Group—DNA methylation tissue**	**Methylation score**					**Polygenic score**				**Total *R*^**2**^ for MS+PGS (%)**
		**CpGs included in MS**	**β_MS_**	**SE_**MS**_**	**P_**MS**_**	**MS *R*^**2**^ (%)**	**β_PGS_**	**SE_**PGS**_**	**P_**PGS**_**	**PGS *R*^**2**^ (%)**	
Birth weight	Adults—Whole blood (*N* = 2,040)	*p* < 10^−1^ pruned	0.063	0.021	0.003	0.394	0.124	0.024	1.96 × 10^−07^^*^	1.525	1.92
	Children—Buccal cells (*N* = 1,070)	*p* < 10^−7^	0.043	0.032	0.172	0.186	0.110	0.030	2.61 × 10^−04^^*^	1.219	1.41
BMI	Adults—Whole blood (*N* = 2,410)	*p* < 10^−7^	0.253	0.026	5.5 × 10^−23^^*^	6.40	0.235	0.022	7.02 × 10^−28^^*^	5.54	11.95
	Children—Buccal cells (*N* = 1,072)	*p* < 10^−1^ pruned	0.017	0.019	0.347	0.030	0.157	0.042	1.7 × 10^−04^^*^	2.451	2.48
Prenatal maternal smoking	Adults—Whole blood (*N* = 720)	*p* < 10^−7^	1.031	0.140	2.4 × 10^−13^^*^	16.886	0.207	0.140	0.139	0.837	17.72
	Children - Buccal cells (N = 547)	*p* < 10^−7^	−0.291	0.187	0.120	1.529	0.283	0.168	0.091	1.093	2.62
Smoking current vs. never	Adults—whole blood (*N* = 1,914)	*p* < 10^−7^ pruned	2.251	0.113	1.3 × 10^−70^^*^	54.41	0.165	0.080	0.042	0.16	54.57
Smoking former vs. never	Adults—whole blood (*N* = 1,938)	*p* < 10^−7^ pruned from current vs. never smoking EWAS	1.216	0.089	1.5 × 10^−34^^*^	15.52	0.159	0.064	0.018	0.51	16.03

*β is the regression coefficient for each term with standard error (SE) and p-value (P). MS R^2^ is the phenotypic variance explained by the MS. PGS R^2^ is the phenotypic variance explained by the PGS. The combined model included the PGS and the best performing MS*.

* *indicates p < 0.0012 in adults and <0.0016 in children*.

### Birth Weight

The birthweight MSs were calculated based on the birthweight EWAS of cord blood samples from neonates (15). The results of GEE showed that none of the MSs was strongly associated with birth weight in adults (*p* < 0.0012) and children (*p* < 0.0016). The pruned blood MS based on 934 CpGs with a *p*-value lower than 1 × 10^−1^ performed better in prediction of birth weight in adults compared with unpruned and other threshold pruned scores, accounting for 0.39% of the variance (*p* = 0.004) (Table 3, Figure 1A). The PGS significantly predicted birth weight in adults (variance explained by PGS = 1.52%, *p* = 1.56 × 10^−7^) (Table 4). The correlation between the whole blood MS and PGS in adults was −0.03 (*p* = 0.182; Supplementary Figure 4A). In the model combining MS and PGS to predict birth weight, the PGS, and blood MS in adults both significantly explained variation in birth weight (variance explained by MS in combined model: 0.39%, *p* = 0.003; by PGS: 1.53%, *p* = 1.96 × 10^−7^ and MS+PGS: 1.92%) (Table 5, Figure 1E).

In children, the best performing score was based on 958 CpGs with a *p*-value lower than 1 × 10^−7^, explaining 0.14% of the variance (*p* = 0.263) (Table 3). The PGS predicted birth weight in children (variance explained by PGS = 1.39%, *p* = 9.67 × 10^−5^) (Table 4). MSs did not add predictive value to PGS in the combined model (Table 5, Figure 1F).

### BMI

Blood MSs for BMI were based on the EWAS by Wahl et al. (12) in blood DNA in adults. These account for a moderate proportion of the variance in BMI in adults (1.5–7.7%). The best performing score explained 7.7% of the variance in BMI (*p* = 9.79 × 10^−27^) and was based on 412 Bonferroni significant CpG sites (Table 3, Figure 1B). The pruned MSs explained less variation in BMI (1.5–4.8% explained variance). The PGS for BMI explained 6.7% of the variance in adults (*p* = 3.43 × 10^−6^; Table 4). The correlation between whole blood MS and PGS was 0.1 in adults (*p* = 1.31 × 10^−6^; Supplementary Figure 4B). In a combined regression model in adults the MS and PGS contributed independently to the prediction of BMI (variance explained by MS in combined model: 6.4%, *p* = 5.46 × 10^−23^, by PGS: 5.5%, *p* = 7.02 × 10^−28^, and MS+PGS: 11.9%) (Table 5, Figure 1E).

In children, the BMI MSs based on buccal methylation data had a considerably lower predictive performance, and none of the scores significantly predicted BMI: the best score in children explained 0.04% of the variance (*p* = 0.276), and was based on 183 pruned CpG sites with a *p*-value lower than 1 × 10^−1^. The PGS explained 3% of the BMI variance (*p* = 2.2 × 10^−5^). MSs did not outperform PGSs in the combined model (Table 5, Figure 1F). Furthermore, removal of children for whom information on BMI was collected more than 3 years before or after DNA collection did not lead to an increase in explained variance (Supplementary Table 4).

### Prenatal Maternal Smoking

In adults, the MSs were based on the EWAS in cord blood from 4,994 newborns (19, 51) and significantly predicted prenatal maternal smoking exposure (Table 3, Figure 1C). The score based on weights of 607 unpruned CpGs at *p* < 1 × 10^−7^ accounted for largest variance of 17.3% of prenatal maternal exposure (*p* = 1.28 × 10^−13^). The pruned MSs performed worse (5.9–7% explained variance). The PGS for maternal smoking around birth did not significantly predict prenatal maternal smoking (variance explained by PGS 1.8%, *p* = 0.05) (Table 4). The correlation between the best performing MS and PGS was 0.06 (*p* = 0.003; Supplementary Figure 4C). The variance explained by MS and PGS in the combined model was slightly lower than predicted by MS alone (variance explained by MS in combined model: 16.9%, *p* = 2.4 × 10^−13^, by PGS: 0.84%, *p* = 0.139 and by MS+PGS: 17.7%) (Table 5, Figure 1E). Maternal smoking scores in buccal methylation data from children, based on the same cord blood discovery EWAS, were not significantly predictive (Table 5, Figure 1F).

### Smoking

The smoking MS in adults were based on the EWAS for never vs. current smokers (18, 51) and were strongly predictive for smoking status. The pruned MSs had a considerably better predictive performance (28–57.5 vs. 21–44% explained variance). The best performing MSs was based on 24 pruned CpGs at *p* < 1 × 10^−7^, and explained 57.5% of variance for current smoking (*p* = 1.25 × 10^−73^) and 16.3% of the variance for former smoking (*p* = 5.44 × 10^−36^) (Table 3, Figure 1D). The PGS for smoking initiation explained 2.8% of the variance in current smoking (*p* = 2.24 × 10^−7^) and 0.9% of the variance of former smoking (*p* = 0.001) (Table 4). The correlation between the PGS of smoking initiation and the best performing MS was 0.14 (*p* = 7.71 × 10^−13^; Supplementary Figure 4D). In the combined prediction model, the MSs outperformed PGSs in the prediction of smoking status, and the PGSs were no longer significant (variance explained by MS in combined model for current vs. never smoking: 54.4%, *p* = 1.3 × 10^−70^, by PGS: 0.16%, *p* = 0.042, and by MS+PGS: 54.6%), indicating that the PGS and MS for smoking do not independently add to prediction of this trait (Table 5, Figure 1E).

## Discussion

We examined if a combined model that includes methylation scores (MS) and polygenic scores (PGS) captures more variance in body size, i.e., birth weight and BMI, and in two exposures, i.e., prenatal maternal smoking exposure and smoking in adulthood, in comparison to the PGS alone. Our results showed that MSs in adults, from blood DNA, predicted BMI, prenatal maternal smoking, and smoking status independent of PGSs, and outperformed PGSs for BMI, prenatal maternal smoking, and smoking status, but not for birth weight. In children, MSs from buccal-cell DNA did not show predictive value in children, but here the tissue in the discovery studies derived from EWASs of cord blood and whole blood DNA methylation profiles.

The most successful MS predictor in our study is for smoking. Blood DNA MS explained up to 57.5% of the variance in current smoking status and 16.3% of the variance in former smoking status. This was substantially better compared to the performance of PGS. Tobacco exposure, both prenatal and current, is a potential environmental exposure that modifies DNA methylation. Several previous studies reported successful application of blood DNA MS created based on weights from an independent discovery EWAS, as we did in the current study (11, 23, 26), based on calculation of indexes (27, 28) or based on machine learning algorithms (25). In line with previous studies, MSs performed better for predicting current vs. never smoking than for former vs. never smoking (25). Most studies of smoking were done on blood DNA methylation. It has been suggested that buccal cell DNA methylation predictors should perform even better (25). Currently, our participants with buccal cell DNA methylation data are too young (methylation data in buccal cells was available for children around 9 years old) to have initiated smoking.

The blood DNA MSs for prenatal maternal smoking, based on cord blood-derived weights from newborns, significantly explained 17.3% of variance in adults. Earlier reports demonstrated that maternal smoking during pregnancy is associated with alterations in offspring blood DNA methylation in newborns (19, 55, 56), children and adolescents (57, 58), and adults several decades after exposure (23, 59). However, the effects of sustained maternal smoking during pregnancy fade away with time, and the predictive accuracy in blood samples from adults is much lower than the accuracy obtained with cord blood samples from newborns (56–58, 60).

We showed that MSs perform better than PGSs in adults for both exposures capturing the effect of smoking, and add value to prediction in combined models. The effects of individual SNPs on behavioral traits such as smoking is small, hence, larger GWAS meta-analyses are required for smoking and maternal smoking to obtain better PGSs. In contrast to PGSs, which capture an individual's genetic predisposition for smoking behavior, MSs capture the effect of exposure to smoking on the methylome. Smoking as an exposure is strongly associated with DNA methylation, and EWAS meta-analyses of smoking and maternal smoking have identified very large numbers of CpGs associated with these traits, allowing for the calculation of fairly reliable MSs, i.e., the EWAS meta-analysis identified over 2,000 significant loci associated with smoking (18), while the currently largest GWAS detected 566 loci associated with smoking initiation (17).

The BMI MS derived in blood samples from adults explained up to 7.7% of the variation in BMI, thereby outperforming the PGS, which explained 6.7% of the variance. Both scores contributed independently to the prediction of BMI in the combined model (12% of explained variance). The performance of adult NTR MSs for BMI was in line with other studies that reported around 10–11% variance explained by MS only in adults (see Figure 1B) and larger variance explained by combined MS and PGS predictors (21, 22). In children with buccal methylation data, a considerably smaller proportion of 2.5% of variation in BMI was explained by the MS and PGS in combined predictor. The lower predictive performance of BMI MSs in children than in adults was also observed in other studies (20, 21), and could be explained by increase of environmental contribution to the trait with age (61) as BMI tends to increase during most of adult life (62). Shah et al. (21) reported that BMI MSs based on an EWAS in adults from the Lothian Birth Cohorts explained 4.9% of variation in adults from the Lifelines DEEP study, but did not account for any BMI variation in adolescents (mean age 14 years) from the Brisbane Systems Genetic Study. Reed et al. (20) observed 10% of BMI variance explained in women in comparison with 1% in children age 7 years and 3% in adolescents age 15 years by MSs calculated on the same set of CpGs from an EWAS in adults.

Birthweight MSs were not strongly predictive in our study, with 0.4 and 0.1% of explained variance in adults and children, respectively, while PGS were significant with 1.5 and 1.4% of explained variance in adults and children, respectively. The PGS for birth weight in NTR was in between the variance explained in previous studies (see Figure 1A): 0.4% in an ALSPAC cohort of 823 newborns (20), 2% in multi-cohort study of 42 thousand twins (53) and in Hyperglycemia and Adverse Pregnancy Outcome (HAPO) study of 1,338 individuals (14), 2.9% in the Northern Finland Birth Cohort (NFBC) of 5,402 individuals and 4.9% in Generation R cohort of 2,357 individuals (14). The discovery EWAS of birth weight (*N* = 8,825 newborns) detected 914 Bonferroni significant CpGs in cord blood (15), suggesting that birth weight does have a large epigenetic signal in cord blood at birth. The cord-blood DNA MS base on weights from adult BMI EWAS accounted for 2% of variance in birth weight (20). According to our knowledge, the performance of birthweight MS based on weights from newborn EWAS has not been previously examined. The low predictive accuracy of the MSs can be caused by the fact the birth weight scores in whole blood and buccal cells were based on an EWAS in cord blood of neonates. Across different tissues and ages, different CpG sites may be associated with birth weight. Another explanation is, that the association between birth weight and DNA methylation fades away with age. Küpers et al. (15) took the 914 significant neonatal blood CpG sites and examined their associations with birth weight in blood samples of adults. No CpG site reached Bonferroni significance in the adults. At present, there are no published EWASs of birth weight based on buccal DNA methylation and no large EWASs of birth weight on blood samples from adults.

The lower predictive accuracy of the buccal cell DNA MSs for all four phenotypes may have several reasons: (1) for some traits there is evidence that DNA methylation signatures increases with age, e.g., BMI, and thus can be not evident at age of 9 years old; (2) unreliability in the phenotype, e.g., prenatal maternal smoking reported participants on their mother's smoking behavior; (3) use of effect sizes from EWASs in cord blood and whole blood methylation data to calculate the scores in buccal cell DNA methylation data. The CpG sites that are predictive for trait/exposure in blood may not be the same CpG sites that are predictive in buccal cells, or the strength of the association may differ across tissues. This last explanation can be tested once more EWASs in buccal cells become available.

The best performing score for each trait will depend on the true number of CpGs associated with the trait and their effect size, the correlation among CpGs, and the power of the discovery EWAS analysis. If the discovery EWAS had full power to detect all CpGs associated with the trait, and there is no large heterogeneity in the effects across cohort, scores created within the same tissue as the discovery EWAS with CpGs based on the most stringent *p*-value threshold (i.e., <1 × 10^−7^) are expected to perform best. More likely, EWASs for these traits did not yet detect all CpGs that are truly associated and larger discovery samples are required to detect CpGs with smaller effects. Therefore, we also examined the performance of scores created based on more lenient *p*-value thresholds. More lenient *p*-value thresholds will potentially add more CpGs to the MS that are truly associated with the phenotype, but which did not yet reach epigenome-wide significance in the discovery EWAS meta-analysis, thereby improving the score. At the same time, inclusion of more CpGs that are not truly associated with the trait and less accurate weights at more lenient thresholds, add more noise to the MS. Pruning was performed to remove correlated CpGs that are redundant (and potentially add noise to scores). The expectation is that if the set of CpGs associated with a trait is correlated (and especially if correlations are strong or abundant), pruning will improve performance of the MS. We found this to be the case, for instance, for blood DNA MS for smoking in adults. For simplicity, we compared two options that have been previously applied in the literature: (1) no pruning at all and (2) a correlation cut-off of 0.1 to select an approximate independent set of CpGs, but we note that the optimal correlation cut-off for pruning may also vary across traits. In adults, pruning reduced the performance of some scores, namely BMI and prenatal maternal smoking, while it improved the performance for birth weight and smoking. Sophisticated methods for MS calculation that model the exact correlation structure between CpGs, as are available for PGS (52), are yet to be developed. In our study, we have selected the best performing score for each trait based on the currently available largest EWAS. With larger discovery EWASs, the optimal selection approach for CpGs is also expected to change.

Birth weight and BMI are physical characteristics, whereas prenatal maternal smoking and own smoking are commonly labeled as exposures and behavioral traits. Birth weight is the least heritable of these traits, while mother's behavior in prenatal maternal smoking consists of an exposure whose genetic contribution is genetically transmittable to offspring, who inherit 50% of mothers' genes. All four complex traits are influenced by genetic variants and environmental factors, although some have argued that behavioral traits are more distal and less directly under biological control than physical traits. Polygenic signals from PGS and MS are composites of signals from different sources that are a result of different combinations of underlying biological processes. Notwithstanding the gap in our understanding about biological processes between the polygenic signals and phenotypes and exposure outcomes, the hypothesis-free approaches from GWAS and EWAS allow for construction of polygenic and methylation scores that have certain predictive accuracy, as demonstrated in research and that have potential for clinical use (10, 63).

The pathways between genome and complex physical and behavioral traits may pass over many different cascades of biological processes in interplay and interaction with environmental factors. PGS and MS can capture different sources of information, from GWASs and EWASs. PGS will capture only genetic vulnerability for a trait, while MSs may capture, in addition to genetic influences on the trait, environmental and stochastic influences and the effect of the trait on the MS.

Pathway analyses indicate that protein products of genes within birthweight-associated regions in GWAS are enriched for diverse processes including insulin signaling, glucose homeostasis, glycogen biosynthesis and chromatin remodeling (14). Birthweight-associates CpGs are among sites that have previously been linked to prenatal maternal smoking and mother's BMI before pregnancy (15). Genes annotated to BMI-associated SNPs are mostly enriched among genes involved in neurogenesis and more generally in the development of the central nervous system (16). Cell type-specific gene expression analysis identified enrichment of brain cell types in BMI (64). These findings suggest that BMI could be considered as a behavioral trait and not only metabolic one. Genes annotated to BMI-associated CpGs play role in adipose tissue biology, insulin resistance, inflammation, as well as metabolic, cardiovascular, respiratory and neoplastic disease (12).

Smoking-initiation-associated genes are involved in dopaminergic and glutamatergic neurotransmission among several regions in the central nervous system related to addictive behavior (17). Many CpGs overlap in both in newborns exposed to prenatal maternal smoking and smoking adults (including cg05575921 (*AHRR*) indicative to smoking exposure in many studies) are implicated in numerous neurological pathways, embryogenesis, and various developmental pathways (51). Unique pathways observed in newborns include xenobiotic-related pathways, cytochrome P450 and uridine-glucoronosyltansferases involved in metabolism of nicotine and other compounds of tobacco smoke (51), and pathways associated to susceptibility to orofacial clefts (19). Unique pathways observed in adults EWAS are enriched for variants associated in GWAS with smoking-related disease, including osteoporosis, colorectal cancers, and chronic obstructive pulmonary disease.

The limitations of our study relate to measurement reliability and the missing data for some phenotypes, which reduced the study power. The largest number of missing data was for prenatal maternal smoking in adults (47%, *N* = 1,133). Women may underreport smoking during pregnancy (24), although in NTR the prevalence of maternal SDP was 19.5 % for the mothers of young twins, which is in line with the prevalence reported in the general Dutch population (48). Birth weight data were missing for many participants who joined the NTR as adults (17%, *N* = 404). In children, there were varying time differences between DNA methylation and BMI measurement. Our sensitivity analysis showed that MS prediction accuracy was not affected by this difference. In the prediction models, we adjusted for age, sex, and cellular composition of samples, hence the predictive performance of MSs reported in this paper is over and above the effects of age, sex, and cellular composition. We recognize that the MSs, and their ability to predict the phenotypes that we study are likely to be impacted by other factors, such as gestational age for birth weight and prenatal maternal smoking, BMI and amount of cigarettes in smoking exposure and vice versa smoking in BMI. Further explorations of potential confounders and mediators will be valuable.

The combination of PGS and MS is a tool to address research questions, such as mediation by DNA methylation of the effect of certain exposures on a trait of interest, where a score based on multiple CpGs may increase the power of such studies compared to a single CpG site.

Lifestyle variables, such as smoking behavior, are often assessed in epidemiologic studies by interviews or questionnaires, and individuals may hide their smoking status or adults may not know if their mother smoked during pregnancy. In such cases, the use of epigenetic profiles can serve as biomarkers and be applied an alternative of survey data (11). Further, the MSs also have potential to be used in risk stratification and disease risk prediction. For example, BMI MSs were shown to predict type 2 diabetes beyond traditional risk factors including BMI and waist–hip ratio (12).

In conclusion, this study illustrates the value of combining PGS with MS for complex trait and exposure prediction. The results of our study provide new insights into the predictive performance of PGS and MS for different traits, across different tissues and ages. Because we analyzed buccal data in NTR children and blood data in NTR adults, the current study could not distinguish between age and tissue as cause for the differences in predictive performance of the scores in the two groups. To make a better distinction between differences caused by age or tissue type, future studies that can create PGS and MS based on both blood and buccal data in children and adults are warranted and ideally both tissues are available for the same individuals. Furthermore, the predictive performance of MSs in blood and buccal methylation data may improve if MSs will be created based on EWA studies performed in the same type of tissue collected at the same age with larger sample size, with other approaches rather than weighted score approach (e.g., machine learning), and the prediction of traits may be further improved by adding information from additional omics levels. Future follow-up studies should investigate relationships between the DNA sequence and DNA methylation in complex traits and exposure outcomes.

## Data Availability Statement

The datasets analyzed in the current study are available from the Netherlands Twin Register on reasonable request. The HumanMethylation450 BeadChip data from the NTR are available as part of the Biobank-based Integrative Omics Studies (BIOS) Consortium in the European Genome-phenome Archive (EGA), under the accession code EGAD00010000887. Analysis code is available upon request from the corresponding author.

## Ethics Statement

The studies involving human participants were reviewed and approved by Central Ethics Committee on Research Involving Human Subjects of the VU University Medical Center, Amsterdam, an Institutional Review Board certified by the U.S. Office of Human Research Protections (IRB number IRB00002991 under Federal-wide Assurance FWA00017598; IRB/institute codes, NTR 03-180). Adult participants or participants' parents/legal guardians provided informed consent.

## BIOS Consortium (Biobank-Based Integrative Omics Study)

### Management Team

Bastiaan T. Heijmans (chair)^1^, Peter A.C. 't Hoen^2^, Joyce van Meurs^3^, Aaron Isaacs^4^, Rick Jansen^5^, Lude Franke^6^.

### Cohort Collection

Dorret I. Boomsma^7^, René Pool^7^, Jenny van Dongen^7^, Jouke J. Hottenga^7^ (Netherlands Twin Register); Marleen MJ van Greevenbroek^8^, Coen D.A. Stehouwer^8^, Carla J.H. van der Kallen^8^, Casper G. Schalkwijk^8^ (Cohort study on Diabetes and Atherosclerosis Maastricht); Cisca Wijmenga^6^, Lude Franke^6^, Sasha Zhernakova^6^, Ettje F. Tigchelaar^6^ (LifeLines Deep); P. Eline Slagboom^1^, Marian Beekman^1^, Joris Deelen^1^, Diana van Heemst^9^ (Leiden Longevity Study); Jan H. Veldink^10^, Leonard H. van den Berg^10^ (Prospective ALS Study Netherlands); Cornelia M. van Duijn^4^, Bert A. Hofman^11^, Aaron Isaacs^4^, André G. Uitterlinden^3^ (Rotterdam Study).

### Data Generation

Joyce van Meurs (Chair)^3^, P. Mila Jhamai^3^, Michael Verbiest^3^, H. Eka D. Suchiman^1^, Marijn Verkerk^3^, Ruud van der Breggen^1^, Jeroen van Rooij^3^, Nico Lakenberg^1^.

### Data Management and Computational Infrastructure

Hailiang Mei (Chair)^12^, Maarten van Iterson^1^, Michiel van Galen^2^, Jan Bot^13^, Dasha V. Zhernakova^6^, Rick Jansen^5^, Peter van 't Hof^12^, Patrick Deelen^6^, Irene Nooren^13^, Peter A.C. 't Hoen^2^, Bastiaan T. Heijmans^1^, Matthijs Moed^1^.

### Data Analysis Group

Lude Franke (Co-Chair)^6^, Martijn Vermaat^2^, Dasha V. Zhernakova^6^, René Luijk^1^, Marc Jan Bonder^6^, Maarten van Iterson^1^, Patrick Deelen^6^, Freerk van Dijk^14^, Michiel van Galen^2^, Wibowo Arindrarto^12^, Szymon M. Kielbasa^15^, Morris A. Swertz^14^, Erik. W van Zwet^15^, Rick Jansen^5^, Peter-Bram 't Hoen (Co-Chair)^2^, Bastiaan T. Heijmans (Co-Chair)^1^.

1. Molecular Epidemiology Section, Department of Medical Statistics and Bioinformatics, Leiden University Medical Center, Leiden, Netherlands

2. Department of Human Genetics, Leiden University Medical Center, Leiden, Netherlands

3. Department of Internal Medicine, ErasmusMC, Rotterdam, Netherlands

4. Department of Genetic Epidemiology, ErasmusMC, Rotterdam, Netherlands

5. Department of Psychiatry, VU University Medical Center, Neuroscience Campus Amsterdam, Amsterdam, Netherlands

6. Department of Genetics, University of Groningen, University Medical Centre Groningen, Groningen, Netherlands

7. Department of Biological Psychology, VU University Amsterdam, Neuroscience Campus Amsterdam, Amsterdam, Netherlands

8. Department of Internal Medicine and School for Cardiovascular Diseases (CARIM), Maastricht University Medical Center, Maastricht, Netherlands

9. Department of Gerontology and Geriatrics, Leiden University Medical Center, Leiden, Netherlands

10. Department of Neurology, Brain Center Rudolf Magnus, University Medical Center Utrecht, Utrecht, The Netherlands

11. Department of Epidemiology, ErasmusMC, Rotterdam, Netherlands

12. Sequence Analysis Support Core, Leiden University Medical Center, Leiden, Netherlands

13. SURFsara, Amsterdam, Netherlands

14. Genomics Coordination Center, University Medical Center Groningen, University of Groningen, Groningen, Netherlands

15. Medical Statistics Section, Department of Medical Statistics and Bioinformatics, Leiden University Medical Center, Leiden, Netherlands

## Author Contributions

VO, VR, JD, and DB: conceptualization and writing—original draft. VO, VR, and JD: prediction analysis. GW, EG, and DB: funding acquisition. FH: sample collection. CB, LL, and GW: phenotype data management. VO and VR: calculation MS. EE, JB, and J-JH: genotyping and genotype data processing. RP and J-JH: calculation PGS. DB and JD: supervision. BIOS consortium: Quality control and analysis pipelines for DNA methylation arrays. All authors: writing—review and editing.

## Conflict of Interest

The authors declare that the research was conducted in the absence of any commercial or financial relationships that could be construed as a potential conflict of interest.

## Publisher's Note

All claims expressed in this article are solely those of the authors and do not necessarily represent those of their affiliated organizations, or those of the publisher, the editors and the reviewers. Any product that may be evaluated in this article, or claim that may be made by its manufacturer, is not guaranteed or endorsed by the publisher.
