# Supplementary material for: Predicting Complex Traits and Exposures From Polygenic Scores and Blood and Buccal DNA Methylation Profiles
Source: Front Psychiatry. 2021 Jul 29;12:688464. doi: 10.3389/fpsyt.2021.688464 (PMC8357987; doi:10.3389/fpsyt.2021.688464)
Supplement: Supplementary file 1 [file Data_Sheet_1.PDF]

Supplemental Information

Predicting complex traits and exposures from polygenic scores and blood and buccal DNA methylation profiles

Veronika V. Odintsova, Valerie Rebattu, Fiona A. Hagenbeek, René Pool, Jeffrey J. Beck, Erik A. Ehli, Catharina E.M. van Beijsterveldt, Lannie Ligthart, BIOS consortium, Gonneke Willemsen, Eco J.C. de Geus, Jouke-Jan Hottenga, Dorret I. Boomsma, Jenny van Dongen

Table S1. Review of methylation scores and combined methylation and polygenic predictors studies of birth weight, BMI, prenatal maternal smoking, and smoking

Trait	Study (First author, year)	Discovery sample for MS	Discovery sample for PGS	Target (prediction) sample	N probes in scores	Performance of MS	Performance of PGS	Performance of combined model (MS+PGS)
Birth weight	**Reed, 2019**	3,743 adults, mean age 67, 79, 70, whole blood, Illumina 450K, Framingham Heart Study and Lothian Birth Cohorts, (Mendelson et al, 2017)	339,224 individuals, European and non-European descent, GIANT BMI study (Locke et al., 2015)	823 children at birth, cord blood, Illumina 450K, ALSPAC	MS: 135 CpGs<br>PGS: 97 SNPs	R^2=2%	R^2=0.4%	NP
BMI	**Hamilton, 2019**	2562 adults, mean age 50, whole blood, Illumina 450K, Generation Scotland participants	339,224 individuals, European and non-European descent, GIANT BMI study (Locke et al., 2015)	892 adults, mean age 69.5, whole blood, Illumina 450K, Lothian Birth Cohort (1936)	MS: 400 CpGs in best predictor (LASSO)	R^2=10%	NP	R^2=18%
				433 adults, mean age 79.1, whole blood, Illumina 450K, Lothian Birth Cohort (1921)	MS: 400 CpGs in best predictor (LASSO)	R^2=6%	NP	NP
	**Shah, 2015**	MS-1: probes and weights from 1,366 adults, mean age 79, 70, whole blood, Illumina 450K, Lothian Birth cohorts (LBC)<br><br>MS-2: probes from 2,377 Framingham Heart Study + weights from LBC<br><br>MS-3: probes and weights from 750 adults, mean age 45, whole blood, Illumina 450K, LifeLines DEEP cohort<br><br>MS-4: probes from 2,377 Framingham Heart Study + weights from LifeLines DEEP	339,224 individuals, European and non-European descent, GIANT BMI study (Locke et al., 2015)	750 adults, mean age 45, whole blood, Illumina 450K, LifeLines DEEP cohort	MS-1 p<1e-07<br>PGS: 96 SNPs	R^2=4.9%	R^2=9.4%	R^2=13%
					MS-2 78 CpGs<br>PGS: 96 SNPs	R^2=7.3%		R^2=16%
				1,366 adults, mean age 79, 70, whole blood, Illumina 450K, LBC	MS-3 p<1e-07<br>PGS: 96 SNPs	R^2=6.9%	R^2=8%	R^2=14%
					MS-4 78 CpGs<br>PGS: 96 SNPs	R^2=11%		R^2=17%
				403 adolescents, twins, mean age 14, whole blood, Illumina 450K, Brisbane Systems Genetic Study (BSGS)	MS-1 p<1e-07<br>PGS: 96 SNPs	R^2=0.1%	R^2=5%	R^2=5%
					MS-2 78 CpGs<br>PGS: 96 SNPs	R^2=3%		R^2=7%
					MS-3 p<1e-07<br>PGS: 96 SNPs	R^2=4%		R^2=7.5%
					MS-4 78 CpGs<br>PGS: 96 SNPs	R^2=5.4%		R^2=8%
	**Reed, 2019**	3,743 adults, mean age 67, 79, 70, whole blood, Illumina 450K, Framingham Heart Study and Lothian Birth Cohorts, (Mendelson et al, 2017)	339,224 individuals, European and non-European descent, GIANT BMI study (Locke et al., 2015)	906 children, mean age 7.5, whole blood, Illumina 450K, ALSPAC	MS: 135 CpGs<br>PGS: 96 SNPs	R^2=1%	R^2=3%	NP
				770 adolescents, mean age 17.4, whole blood, Illumina 450K, ALSPAC		R^2=3%	R^2=5%	NP

				792 pregnant mothers, mean age 28.9, whole blood, Illumina 450K, ALSPAC		R^2=2%	R^2=2%	NP
				726 mothers, mean age 47.7, whole blood, Illumina 450K, ALSPAC		R^2=10%	R^2=2%	NP
Prenatal Maternal Smoking	Reese, 2017	1,057 newborn, cord blood, Illumina 450K, Norwegian Mother and Child Cohort Study (MoBa) subcohort (training set)	NP	221 newborns, cord blood, Illumina 450K, MoBa subcohort (testing set)	LASSO tested 195 top significant CpGs. Combined cotinine-based and self-report based sustained smoking score 28 CpGs	Not reported.<br>AUC 0.96 training set, 0.90 testing set; accuracy 96%, sensitivity 80%, specificity 98%	NP	NP
	Richmond, 2018 (prenatal smoking exposure in women)	Newborn EWAS based MS: 6685 newborns, cord blood, Illumina 450K, PACE (Joubert et al., 2016)<br>Children EWAS based MS: 3187 children, mean age 6.8, whole blood, Illumina 450K, CHARGE (Joubert et al., 2016)	NP	754 women 30 years old, whole blood, Illumina 450K, ALSPAC<br>Replication: 656 women 18 years later, 48 years old and 230 men, 53 years old	Newborn EWAS based 568 CpGs<br><br>Children EWAS based<br>19 CpGs	Not reported. Newborn EWAS based score AUC 0.69 (95% CI 0.67, 0.73). Children EWAS based score AUC 0.72 (95% CI 0.69, 0.76).<br>MS where higher in individuals who were exposed to prenatal smoking compared with non-smokers who were not exposed prenatally.	NP	NP
Smoking	Richmond, 2018 (smoking)	9389 adults, whole blood, Illumina 450K (Joehanes et al, 2016)	NP	754 women 30 years old, whole blood, Illumina 450K, ALSPAC<br>Replication: 656 women 18 years later, 48 years old and 230 men, 53 years old	2623 CpGs<br>Score predicts whether the mothers had smoked during pregnancy	Not reported. AUC in women 0.88 (95% CI 0.85, 0.91)	NP	NP
	Bollepalli, 2019	474 adults, Finnish cohort (training set)	NP	5 testing datasets:<br>408 adults, Finnish Twin Cohort, 687 adults, EIRA study, 464 adults CARDIOGENICS consortium, whole blood, Illumina 450K; and publicly available buccal and PBMC's datasets	LASSO tested 52,421 probes resulting in classifier of 121 CpGs EpiSmokEr (Epigenetic Smoking status Estimator)	Not reported. In blood datasets: Self-reported current: 81% sensitivity, 85% specificity<br>Self-reported never smokers: 94% sensitivity, 57% specificity<br>Self-reported former smokers: 18% sensitivity, 96% specificity<br><br>In buccal cells<br>Self-reported current: 95% sensitivity, 97%	NP	NP

						specificity. Former: 37% specificity		
	Elliott, 2014	1793 adults (current, former, never smoking), (Zeilinger et al, 2013)	NP	189 adults, SABRE (population-based cohort including South Asian and European origin)	183 CpGs	Not reported. One unit increase in smoking score was associated with 0.54 increase in number of cigarettes smoked per day in South Asians, and was not associated with number of cigarettes smoked per day in Europeans	NP	NP
	Sugden, 2019	9389 adults, whole blood, Illumina 450K (Joehanes et al, 2016)	NP	Dunedin Study, N=1037<br>E-Risk Study N=2,232	2623 CpGs in SmPEGS (Smoking methylation PolyEpigenetic Score)	Not reported. AUC range from 0.77-0.93	NP	NP
	Zhang et al., 2016	374 adults Epic-Turin, Cancer and Nutrition Cohort<br>(Shenker et al., 2013) and 1793 adults (Zelinger et al., 2013), whole blood, Illumina 450K	NP	9,949 adults, age 50–75 years, whole blood, Illumina 450K	2 CpGs (cg05575921 and cg06126421)	Not reported. Regression for smoking: dose-response curves only for each CpG separately. MS prediction was performed for other traits: strong associations with all-cause, cardiovascular, and cancer mortality.	NP	NP
	Nwanaji-Enwerem et al, 2019	Preselected CpGs from Gao et al, 2015 and DNAm-age studies (whole blood, Illumina 450K)	NP	120 men, buccal cells, Illumina 450K	66 CpGs in Smoking index (mean β value (μ_c) and standard deviation (σ_c))	Not reported. Sensitivity 90% (smokers), specificity 85% (nonsmokers),<br>AUC 0.92	NP	NP

NP=not performed; PBMC=peripheral blood mononuclear cells

Note: some studies performed multiple scores for multiple phenotypes and appear several times in the table. Studies included in Figures 1 are indicated in bold

Table S2. Overview of Discovery studies for methylation scores

Trait	Discovery study (First author, Year)	Discovery Sample	Discovery Sample Size	Threshold for inclusion of CpGs	Adult Methylation Scores (blood DNA, Illumina 450 K)			Children Methylation Scores (buccal cell DNA, Illumina EPIC)		
					N CpGs in discovery summary stat	N CpGs in unpruned MS	N CpGs pruned MS	N CpGs in discovery summary stat	N CpGs in unpruned MS	N CpGs pruned MS
Birth weight	Kupers 2019	Newborns, cord blood, Illumina 450K	8,825	$p<10^{-1}$	78,489	72,570	934	78,489	72,205	184
				$p<10^{-5}$	2,423	2,274	30	2,423	2,249	13
				$p<10^{-7}$	1,029	963	18	1,029	958	9
BMI	Wahl 2017	Adults, whole blood, Illumina 450K	5,387	$p<10^{-1}$	63,183	55,653	671	63,183	55,279	183
				$p<10^{-5}$	1,168	1,067	13	1,168	1,079	13
				$p<10^{-7}$	450	412	6	450	422	6
Prenatal Maternal Smoking	Sikdar 2019	Newborns, cord blood, Illumina 450K	4,994	$p<10^{-1}$	85,787	76,531	962	85,787	76,146	187
				$p<10^{-5}$	1,694	1,581	33	1,694	1,571	16
				$p<10^{-7}$	648	607	16	648	606	9
Smoking Current vs never	Sikdar 2019	never/current smokers, whole blood, Illumina 450K	9,389	$p<10^{-1}$	110,546	98,972	913	NA	NA	NA
				$p<10^{-5}$	12,519	11,433	37	NA	NA	NA
				$p<10^{-7}$	7,604	6,938	24	NA	NA	NA
Smoking Former vs never	Joehanes 2016	never/former smokers, whole blood, Illumina 450K	13,474	FDR sign	2,568	2,330	20	NA	NA	NA

Illumina 450 K = Illumina450K Beadchip (Illumina Inc., CA, USA), Illumina EPIC = Infinium MethylationEPIC BeadChip Kit (Illumina, San Diego, CA, USA)

Table S3. Overview of Discovery studies for Polygenic scores

Trait	Discovery study	Discovery Sample	Discovery Sample Size	SNP fraction	N SNPs in Discovery Summary Statistics	N SNPs in PGS
Birth weight	UK Biobank (data field 20022) [http://www.nealelab.is/uk-biobank/]	population-based	280,250	0.01	13,791,467	9,173,894
BMI	Yengo et al, 2018	population-based	652,099*	0.5	2,502,038	2,018,248
Prenatal Maternal Smoking	UK Biobank (https://atlas.ctglab.nl/ukb2_sumstats/ f.1787.0.0_logistic.EUR.sumstats.MACfilt.txt.gz)	population-based	331,862	0.5	10,456,754	9,999,200
Smoking	Liu et al, 2019	smoking initiation	625,536**	0.5	15,517,156	5,972,355

*We obtained summary statistics from the meta-analysis without NTR and no 23me
**We obtained summary statistics from the meta-analysis without NTR
SNP fraction = fraction of causal SNPs specified in LDpred (see Methods).

Figure S1. Traits/exposures distribution

Adults

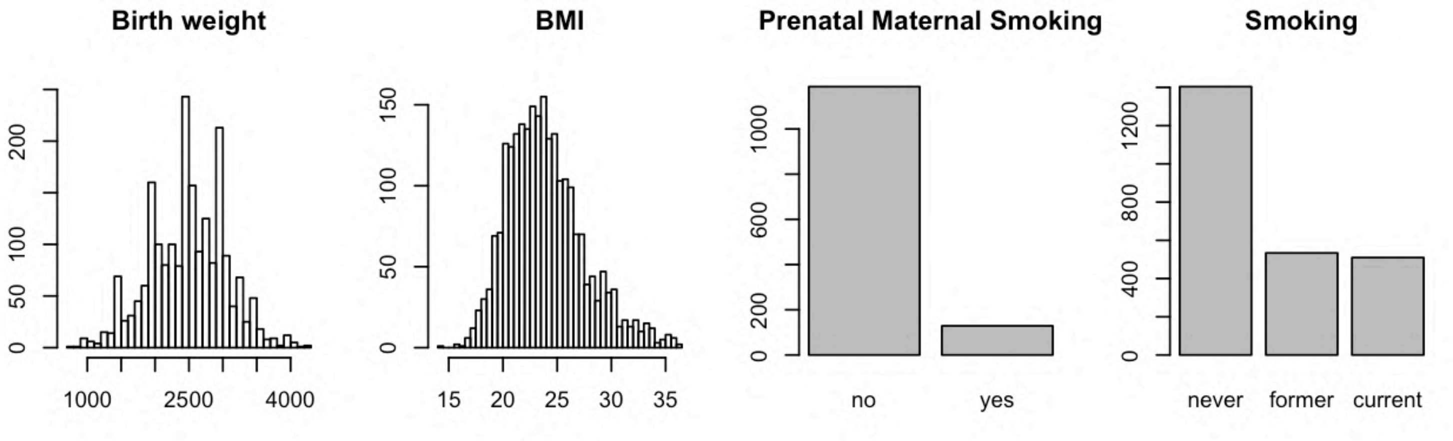

Children

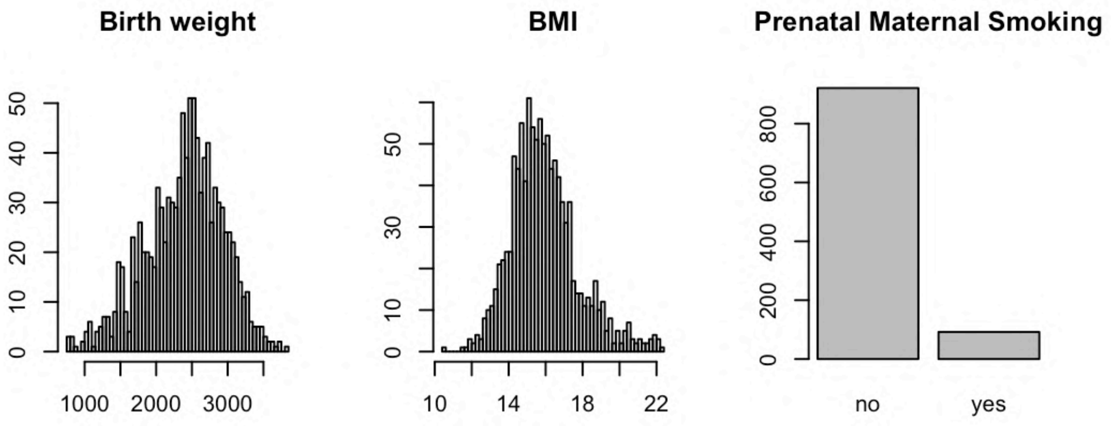

Figure S2. Distribution of MS and PGS in NTR adults

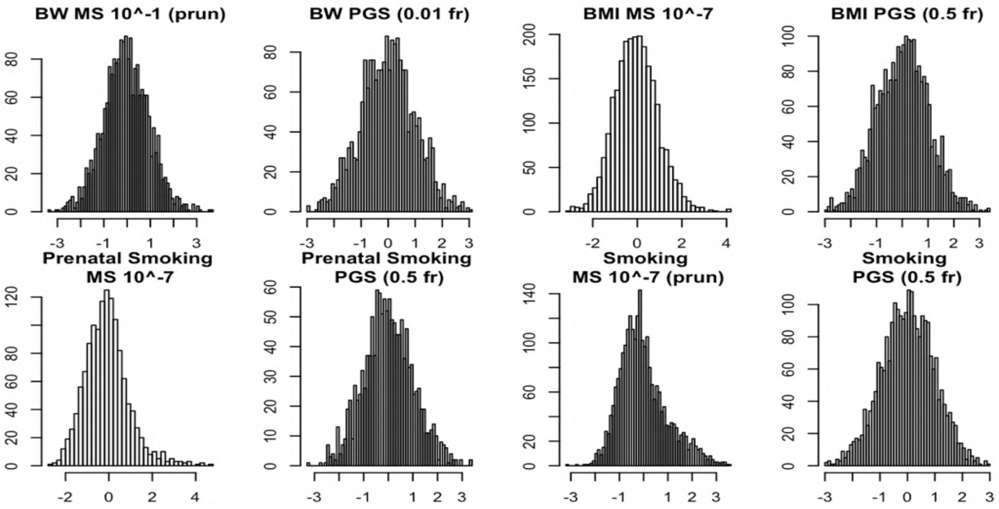

Figure S3. Distribution of MS and PGS in NTR children

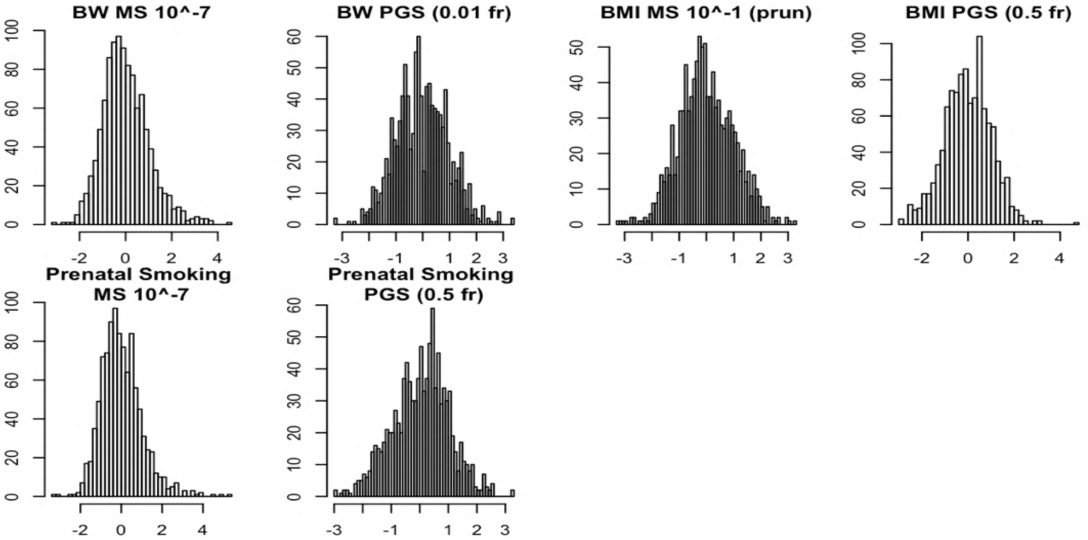

BW=birth weight; BMI=body mass index; prun=pruned; fr= fraction of causal SNPs specified in LDpred (see Methods).

Figure S4. Correlations of trait/exposure with predictors in adults

A)

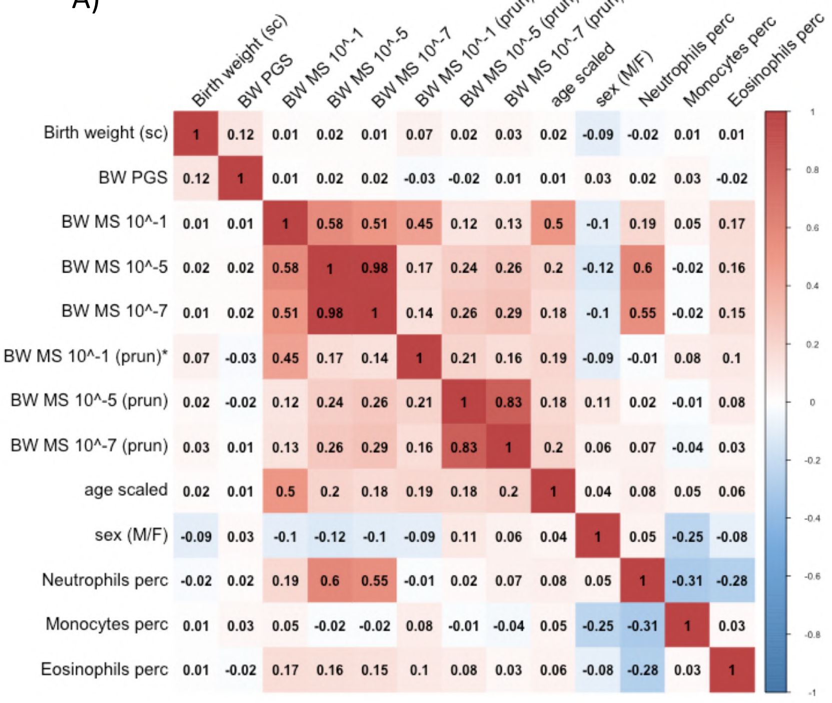

$r^{PGS\text{-}MS^*}$=-0.027, p=0.182

B)

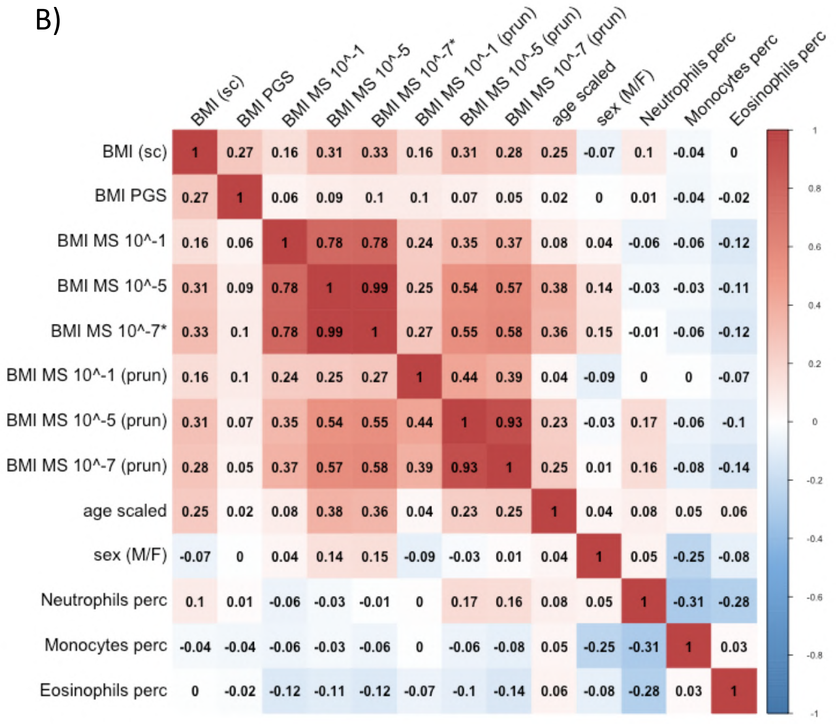

$r^{PGS\text{-}MS^*}$=0.098, p=1.31x10^{-6}

C)

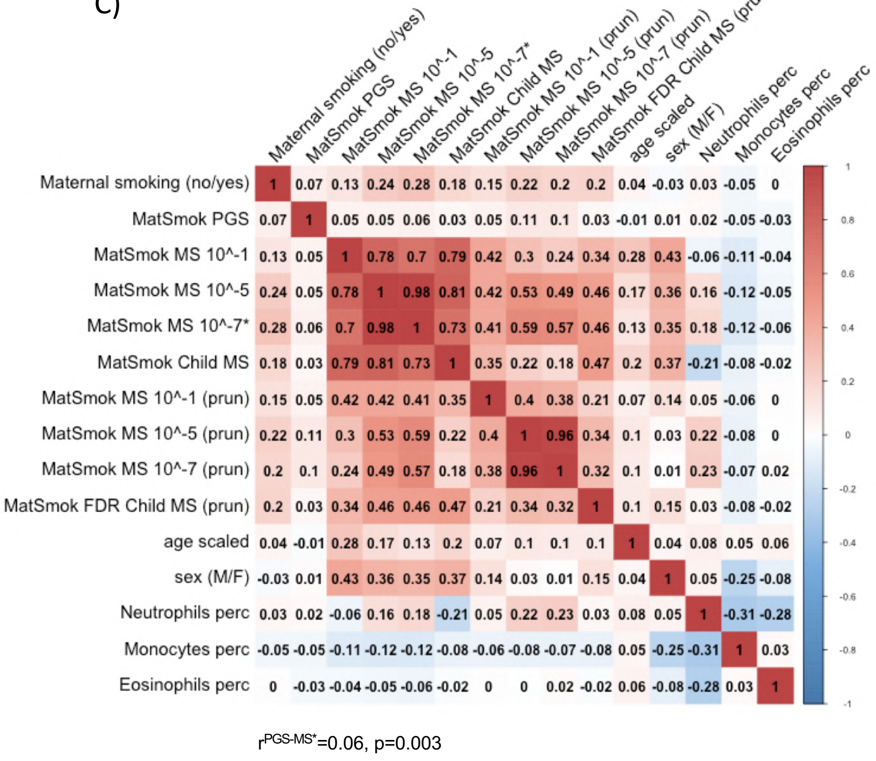

D)

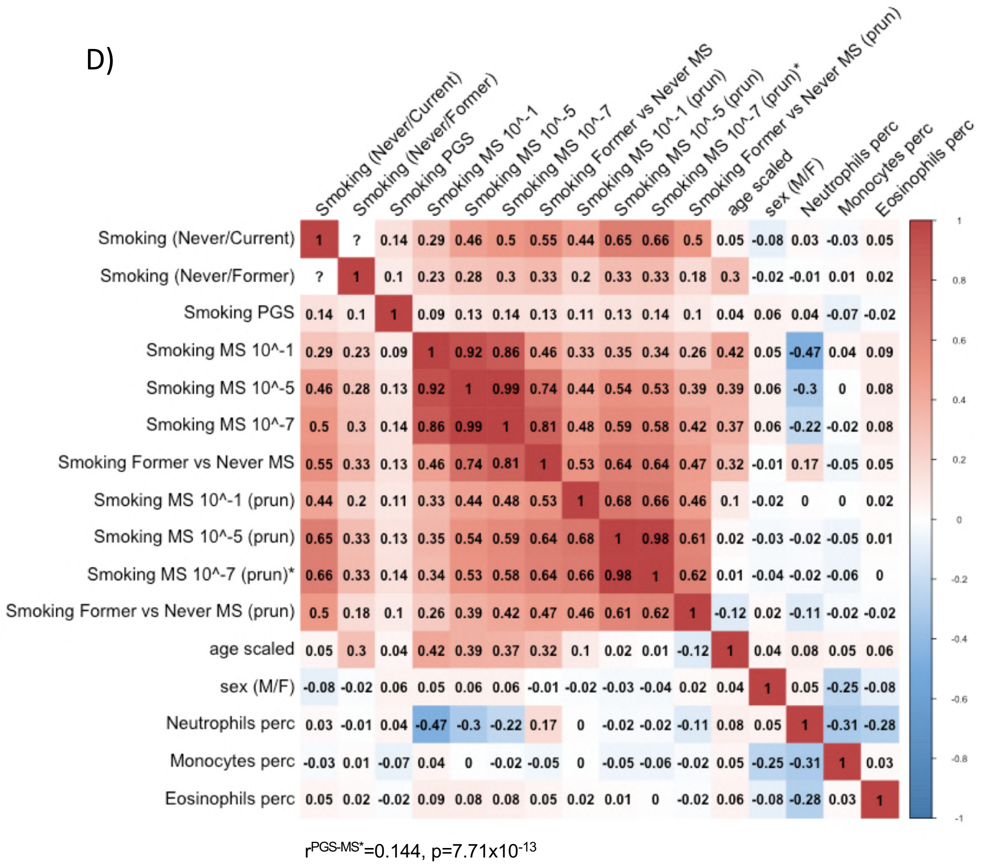

A) birth weight, B) BMI, C) prenatal maternal smoking, D) smoking
MS=methylation score, PGS=polygenic score, sc = scaled, prun = pruned, perc = percentage.
N = 2431
* MS with lowest p-value in model 1

Figure S5. Correlations of trait/exposure with predictors in children

A)

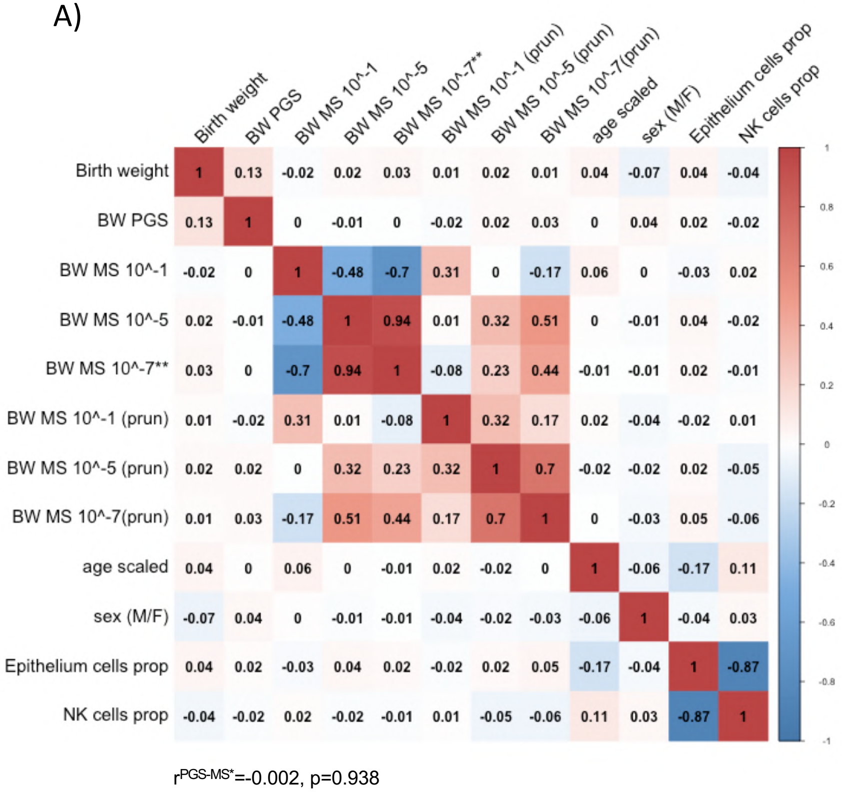

B)

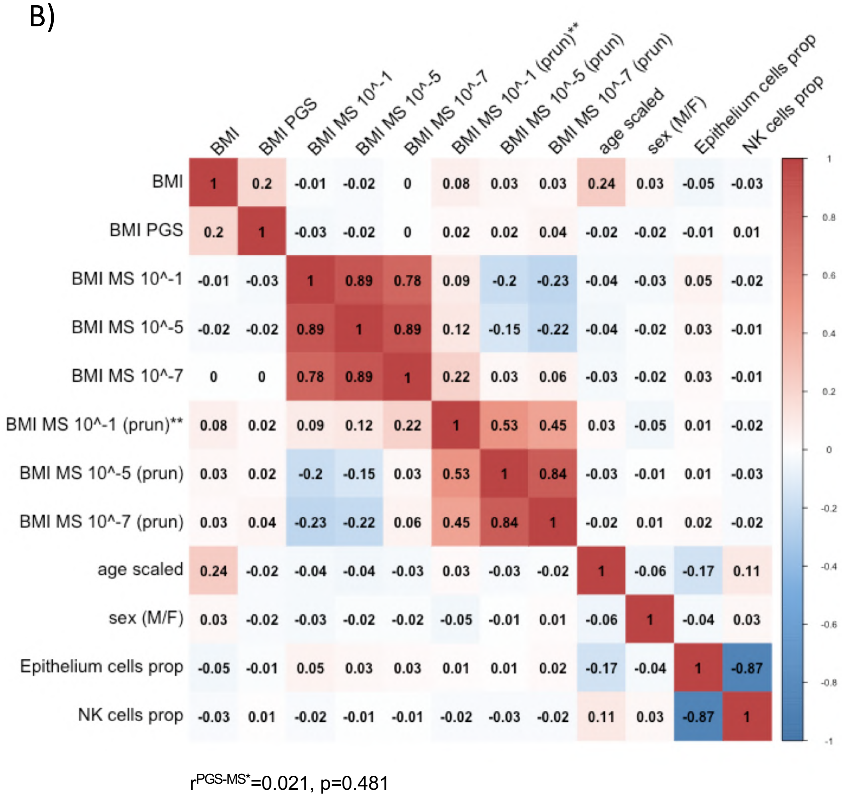

C)

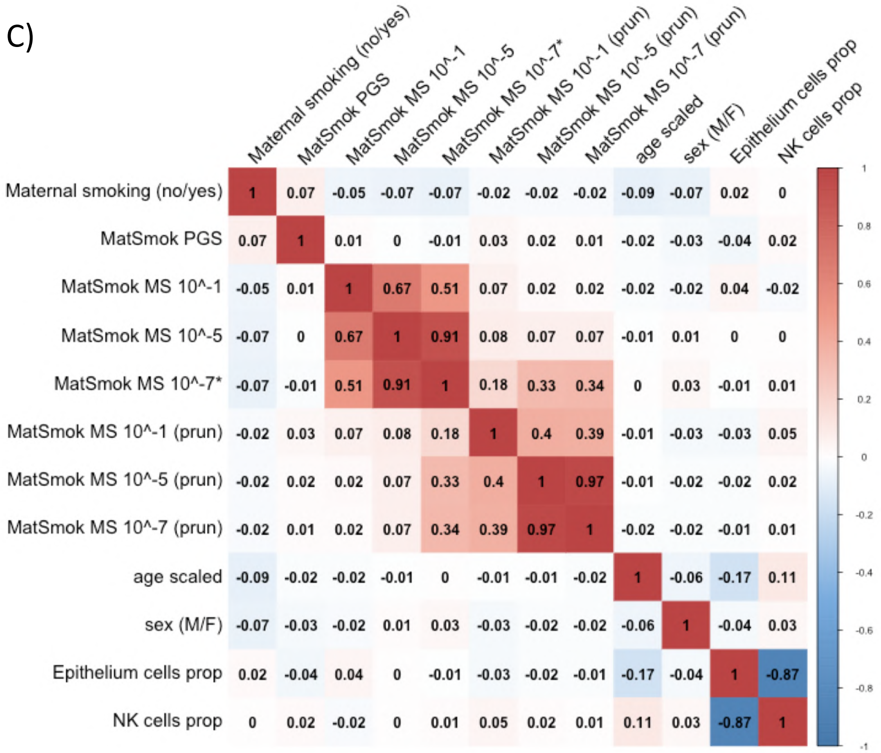

$r^{PGS\text{-}MS*}$=-0.011, p=0.722

A) birth weight, B) BMI, C) prenatal maternal smoking
MS=methylation score, PGS=polygenic score, sc = scaled, prun = pruned, perc = percentage.
N = 1128
* MS with lowest p-value in model 1

Table S4. Sensitivity analysis for children BMI (maximum 3-year period between BMI measurement and DNA collection)

<table>
<tr><th rowspan="2"></th><th colspan="5">Methylation Score</th><th colspan="4">Polygenic Score</th><th rowspan="2">Combined predictor MS+PGS R2 (%)</th></tr>
<tr><th>Threshold for inclusion of CpGs in MS</th><th>β_{MS}</th><th>SE_{MS}</th><th>P_{MS}</th><th>MS R^2 (%)</th><th>β_{PGS}</th><th>SE_{PGS}</th><th>P_{PGS}</th><th>PGS R^2 (%)</th></tr>
<tr><td rowspan="6">One-score models</td><td>p<10-1</td><td>0.004</td><td>0.022</td><td>0.863</td><td>0.00</td><td rowspan="6">0.197</td><td rowspan="6">0.051</td><td rowspan="6">0.0001</td><td rowspan="6">3.89</td><td rowspan="6">NA</td></tr>
<tr><td>p<10-5</td><td>-0.010</td><td>0.023</td><td>0.656</td><td>0.01</td></tr>
<tr><td>p<10-7</td><td>-0.006</td><td>0.024</td><td>0.806</td><td>0.00</td></tr>
<tr><td>p<10-1 pruned*</td><td>0.030</td><td>0.024</td><td>0.199</td><td>0.09</td></tr>
<tr><td>p<10-5 pruned</td><td>0.029</td><td>0.024</td><td>0.226</td><td>0.08</td></tr>
<tr><td>p<10-7 pruned</td><td>0.019</td><td>0.023</td><td>0.423</td><td>0.04</td></tr>
<tr><td>Combined model</td><td>p<10-1 pruned</td><td>0.028</td><td>0.023</td><td>0.218</td><td>0.08</td><td>0.194</td><td>0.051</td><td>0.0001</td><td>3.74</td><td>3.82</td></tr>
</table>

N=748

Note: β is the regression coefficient for each methylation score (MS) with standard error (SE) and p-value (P). MS R^2 is the phenotypic variance explained by the MS.
One-score models include either MS or PGS in the prediction, and combined model includes both MS and PGS. * indicate methylation score with lowest p-value
